# Dynamic Ion Gels from the Complex Coacervation of Oppositely Charged Poly(ionic liquid)s

**DOI:** 10.1021/acsmacrolett.4c00253

**Published:** 2024-07-11

**Authors:** Malak Alaa Eddine, Daniil R. Nosov, Luiz Fernando Lepre, Anatoli Serghei, Daniel F. Schmidt, Damien Montarnal, Alexander S. Shaplov, Eric Drockenmuller

**Affiliations:** ‡ Université Claude Bernard Lyon 1, CNRS, Ingénierie des Matériaux Polymères, UMR 5223, Lyon, F-69003, France; § Luxembourg Institute of Science and Technology (LIST), 5 Avenue des Hauts-Fourneaux, L-4362 Esch-sur-Alzette, Luxembourg; ∥ Department of Physics and Materials Science, University of Luxembourg, 2 Avenue de l’Université, L-4365 Esch-sur-Alzette, Luxembourg; ⊥ Université Claude Bernard Lyon 1, CPE Lyon, CNRS, Catalyse, Polymérisation, Procédés et Matériaux, UMR 5128, Lyon, F-69003, France

## Abstract

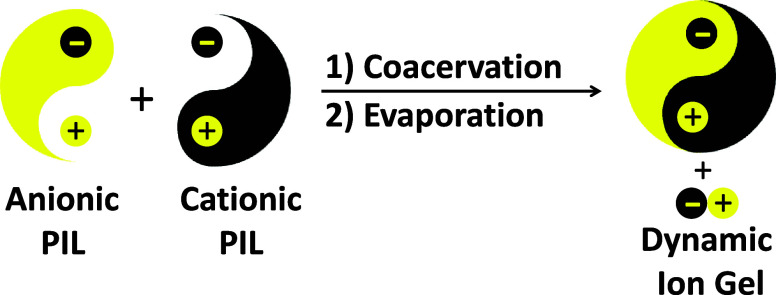

A cationic poly(ionic liquid) (PIL) with pendent butyl imidazolium cations and free bis(trifluoromethylsulfonyl)imide (TFSI) anions and an anionic PIL with pendent TFSI anions and free 1-butyl-3-methylimidazolium cations are synthesized by postpolymerization chemical modification and reversible addition–fragmentation chain-transfer radical copolymerization, respectively. Upon mixing solutions of these two PILs in acetone with stoichiometric amounts of ion pairs, ionic exchanges induce coacervation and, after solvent evaporation, lead to the formation of a dynamic ion gel (DIG) and the concomitant release of free [1-methyl-3-butyl imidazolium]TFSI ionic liquid (IL). A comparison of thermal (*T*_g_), ion conducting (σ_DC_), and viscoelastic (elastic moduli (*G*′)) properties for DIGs and their parent polyelectrolytes, as well as extracted and IL-doped DIGs, demonstrates the formation of ionic cross-links and the ability to easily produce polymer electrolytes with enhanced ionic conductivity (σ_DC_ up to 4.5 × 10^–5^ S cm^–1^ at 30 °C) and higher elastic moduli (*G*′ up to 4 kPa at 25 °C and 1 rad s^–1^), making them highly desirable in many electrochemical applications, including supercapacitors, soft robotics, electrochromic devices, sensors, and solar cells.

Interest has shifted over time from flammable liquid electrolytes to safer solid polymer electrolytes (SPE) and poly(ionic liquid)s (PILs) given their potential to revolutionize electrochemical devices by combining the ion transport properties of ionic liquids (ILs) with the mechanical stability and processability of polymers. Ideal PILs should possess high dimensional stability and ionic conductivity (>10^–5^ S cm^–1^ at 25 °C).^1−3^ High dimensional stability usually requires PILs to have a high glass transition temperature (*T*_g_) and/or possess partial crystallinity, while high ionic conductivity demands low *T*_g_ amorphous polymers to facilitate segmental motion and promote fast ion transport.^2,4^ One means of maintaining high ionic conductivity in SPEs is by forming ion gels, i.e., soft composite polymers consisting of an IL embedded in a polymeric matrix.^4−6^ Common ion gels contain 40–50 wt % of IL,^7^ affording ionic conductivity levels of 10^–4^–10^–6^ S cm^–1^ at 25 °C and preserving to some extent the mechanical properties attributed to neat PILs.^4^ However, further increases in IL content (≥60 wt %) and ionic conductivity (to 10^–3^ S cm^–1^ at 25 °C) lead to loss of mechanical stability, making ion gels prone to IL leakage both over time and as a function of external conditions (i.e., in compression^8^).

Another approach involves the formation of polyelectrolyte complexes (PECs) or coacervates between oppositely charged macromolecules with improved viscoelastic properties and/or processability.^9^ The term “PEC” was introduced by Bungenberg de Jong and Kruyt to distinguish coacervation of two polyelectrolytes from that of a polyelectrolyte and a small molecule.^10,11^ An example of PEC formation is the coacervation of poly(3,4-ethylenedioxythiophene) and poly(4-styrenesulfonate) (PEDOT:PSS), enhancing mechanical properties and film forming ability of otherwise brittle and insoluble PEDOT.^12^ PECs based on PSS and poly(diallyldimethylammonium) chloride obtained in aqueous media were used in biomedical applications such as deep tissue bonding, bone fixation, scaffold coatings and drug encapsulation.^13^ Nevertheless, to the best of our knowledge, this method was never used to improve ionic conductivity, and the salt formed during the coacervation of PECs (e.g., CsCl, NaCl, ...) is generally washed out prior to application. It is also noteworthy that PECs are almost exclusively based on water-soluble polyelectrolytes. An earlier example in nonaqueous media was reported by Hayward and co-workers.^14^ They investigated the coacervation of a polycation containing 1-(2-acryloyloxyethyl)-3-butylimidazolium bis(trifluoromethanesulfonyl)imide units and a polyanion containing 1-ethyl-3-methylimidazolium 3-sulfopropyl acrylate units in high dielectric constant (ε_r_) organic solvents: 2,2,2-trifluoroethanol (TFE, ε_r_ = 27.0) and hexafluoro-2-propanol (HFIP, ε_r_ = 16.7). They examined the effect of additional IL on the system. In the absence of added IL, both systems exhibited two physical states of the polymer-rich phase: solid-like precipitates and fluid-like coacervates. The solvent significantly influenced the formation of a homogeneous solution as the salt concentration increased, with HFIP showing a greater tolerance for IL addition vs TFE.

Thus, with the aim to enhance both mechanical stability and ionic conductivity, this work describes an alternative approach relying on the coacervation of complementary PILs having ion pairs with identical structural features (Scheme 1). A new class of polyelectrolyte complexes termed Dynamic Ion Gels (DIGs) was prepared from the solution mixing of complementary cationic and anionic PILs bearing highly delocalized and ion conductive imidazolium and bis(trifluomethylsulfonyl)imide (TFSI) moieties. The gelation process proceeded via the formation of dynamic ionic cross-links through ion metathesis and in situ generation of a highly ion conducting IL (i.e., [1-methyl-3-butyl imidazolium]TFSI, IL). The formation of ionic cross-links in DIGs was first demonstrated by quantifying IL release by both liquid extraction and quantitative ^19^F NMR spectroscopy. Both experiments showed that ∼90% of the ion pairs of PIL^+^ and PIL^–^ undergo interchain ion metathesis to form DIGs through coacervation. Furthermore, the relationship between ionic conductivity and viscoelastic properties has been established for DIGs containing from 0 to 33 wt % of IL. In particular, the as-formed DIGs show superior viscoelastic performance (elastic moduli, *G*′) and ionic conductivity vs the parent polyelectrolytes. In addition, the balance between these important applicative properties was easily tuned either by IL addition (thus, enhancing ion transport) or by IL extraction (thus, enhancing mechanical performance). In this way, the overall properties profile of these innovative SPEs may be specifically adjusted for various electrochemical applications.

**Scheme 1:**
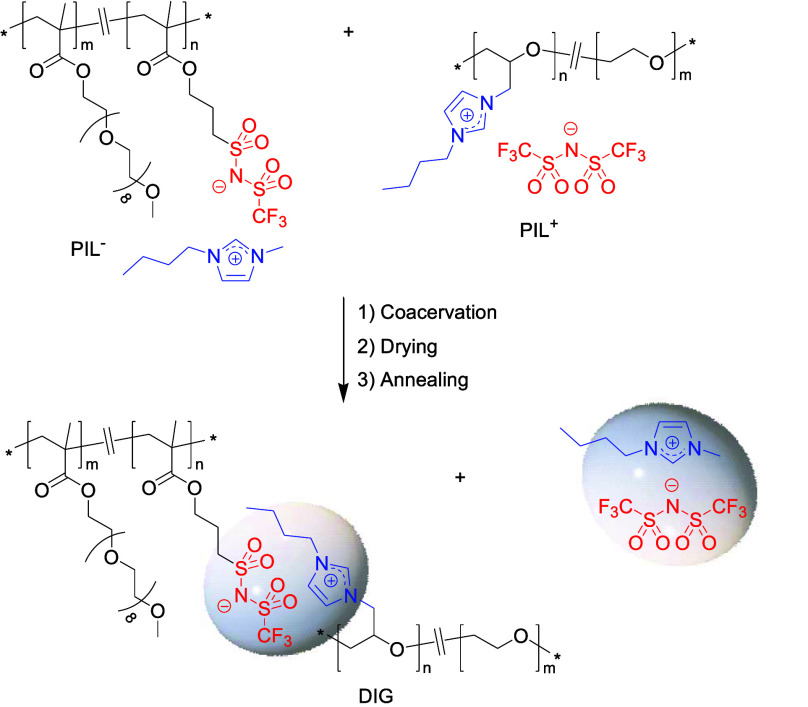
Formation of Dynamic Ion Gels from the Complex Coacervation of Oppositely Charged Poly(ionic liquid)s

Polyether/imidazolium/TFSI-based cationic PIL^+^ was prepared via *N*-alkylation of *N*-butylimidazole by the pendent chloromethyl groups of a commercially available functional copolyether having a 1:1 molar ratio of ethylene oxide and epichlorohydrin followed by ion exchange with lithium bis(trifluoromethylsulfonyl)imide (Scheme S1).^15^ A methacrylate-based ionic liquid monomer (ILM) was synthesized by ion exchange between lithium 1-[3-(methacryloyloxy)propylsulfonyl]-1-(trifluoromethanesulfonyl)imide and 1-butyl-3-methylimidazolium bromide (Scheme S2).^16^ The polymethacrylate/imidazolium/TFSI-based anionic PIL^–^ was then obtained by reversible addition–fragmentation chain transfer (RAFT) copolymerization of a 1:1 molar ratio of ILM and poly(ethylene glycol) methyl ether methacrylate (PEGM) using 4-cyano-4-(phenylcarbonothioylthio)pentanoic acid as chain transfer agent (Scheme S3).^17−20^ The structures of PIL^+^ (Figures S1–S4), ILM (Figures S6–S9) and PIL^–^ (Figures S10–S13) were confirmed by ^1^H, ^13^C, and ^19^F NMR and FTIR spectroscopies and elemental analysis. The number-average molar masses (*M*_n_ = 110 and 72 kg mol^–1^) and chain dispersities (*Đ* = 3.45 and 1.50) for PIL^+^ and PIL^–^, respectively, were determined by SEC (Figures S5 and S14). These differences together with variations in the size and flexibility of the pendent groups lead to distinct viscoelastic behaviors: at ambient temperature, while PIL^+^ is highly entangled and shows rubbery behavior, PIL^–^ is a viscous cold flowing fluid (Figures 1 and S15). Their thermal properties (Table 1) were evaluated by differential scanning calorimetry (DSC, Figure S16) and thermogravimetric analysis (TGA, Figure S17). For PIL^+^, the location of the ion pairs near the flexible polyether backbone results in a higher glass transition temperature (*T*_g_) of −30 °C. Due to the presence of longer, more mobile oligoether side chains acting as internal plasticizers, PIL^–^ exhibits a lower *T*_g_ value of −46 °C. Due to the presence of aliphatic polyether segments, both PIL^+^ and PIL^–^ exhibit moderate thermal stability with *T*_onset_ temperatures of 290 and 240 °C, respectively.

**Figure 1:**
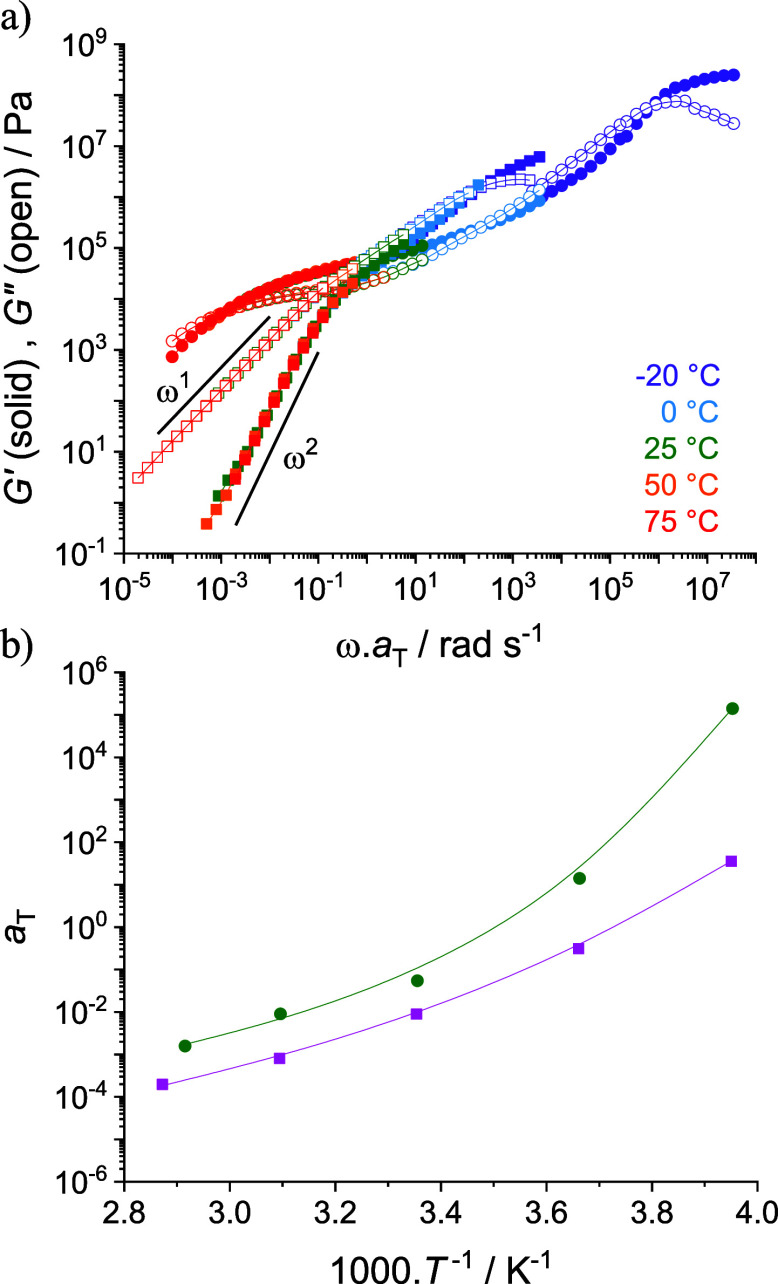
(a) TTS master curves of PIL^+^ (circles) and PIL^–^ (squares) obtained by performing SAOS rheology at temperatures ranging from −20 to 75 °C and referenced at *T*_0_ = *T*_g_ + 40 K. (b) Corresponding *a*_T_ shift factors and WLF best fits at *T*_0_ = *T*_g_ + 40 K for PIL^+^ (green circles) and PIL^–^ (pink squares).

**Table 1:** Physical and Ion Conducting Properties of PILs, IL, and DIGs

sample	IL content (wt %)	*T*_g_^a^ (°C)	*T*_onset_^b^ (°C)	σ_DC_ at 30 °C^c^ (S cm^–1^)
PIL^+^	0	–30	290	6.4 × 10^–6^
PIL^–^	0	–46	240	9.9 × 10^–6^
IL	100	–87^d^	320	4.1 × 10^–3^
DIG_IL_	33^e^	–49	245	4.5 × 10^–5^
DIG	23.8^e^/24.6^f^	–39	250	1.2 × 10^–5^
DIG_ext_	0	–30	220	9.0 × 10^–8^

a Determined by DSC.

b *T*_onset_ determined by TGA.

c Determined by BDS.

d Reported by MacFarlane and Forsyth.^28^

e Determined by quantitative ^19^F NMR.

f Determined by gravimetry after solvent extraction.

The viscoelastic properties of PIL^+^ and PIL^–^ were investigated by small amplitude oscillatory shear (SAOS) rheology from −20 to 75 °C. To facilitate comparisons, master curves were built through time–temperature superposition (TTS) using a reference temperature of *T*_0_ = *T*_gDSC_ + 40 K (Figure 1a). PIL^+^ exhibits a rubbery pseudoplateau spanning ca. 3 decades of frequency, typical of entangled polymer chains. The slope of the rubbery pseudoplateau is due to the high chain dispersity of PIL^+^ (*Đ* = 3.45). The average molar mass between entanglements (*M*_e_ = 53 kg mol^–1^) was calculated using eq 1:
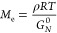
1with ρ the melt density, *R* the gas constant, *T* the absolute temperature, and *G*_*N*_^0^ the plateau value of the storage modulus (taken at *G*_*N*_^0^ = *G*′(ω)_min(tan δ)_ ≈ 60 kPa).^21,22^ Conversely, PIL^–^ displays viscoelastic behavior typical of unentangled polymer chains with terminal regimes evidenced by the evolution of loss (*G*″) and storage (*G*′) moduli following ω^1^ and ω^2^ slopes, respectively. The shift factors (*a*_T_) used in the TTS of PIL^+^ and PIL^–^ obey a WLF law (Figure 1b and Table S1).

The bulk anhydrous ionic conductivity (σ_DC_) of PIL^+^ and PIL^–^ was measured by broadband dielectric spectroscopy (BDS) from 110 to −50 °C (Figures 2, S18, and S19 and Table 1). Due to correlations between ion transport and polymer chain dynamics, the evolutions of σ_DC_ with temperature were fitted using the Vogel–Fulcher–Tammann (VFT) eq 2:
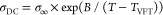
2with σ_∞_ the ionic conductivity in the limit of high temperatures, *B* is a fitting parameter related to the activation energy of ionic conduction, and *T*_VFT_ is the Vogel temperature, which is generally ca. 50 K below *T*_g_ (Table S2). σ_DC_ of PIL^–^ is slightly higher than that of PIL^+^ (σ_DC_ at 30 °C = 9.9 × 10^–6^ and 6.4 × 10^–6^ S cm^–1^, respectively), as observed previously for polyanionic and polycationic analogues.^4,23^ Despite a lower charge density (0.93 vs 1.98 mmol·g^–1^ for PIL^–^ and PIL^+^,^24^ respectively), the higher ionic conductivity of PIL^–^ can be explained by its lower *T*_g_ (−46 vs −30 °C for PIL^–^ and PIL^+^, respectively), the presence of the oligo(ethylene glycol) side chains promoting ion dissociation as well as the longer spacer between the polymer backbone and the tethered ion pairs.^4,25−27^ While lower than highest ion conducting PILs reported to date (Table S3), PIL^+^ and PIL^–^ exhibit levels of ionic conductivity that place them among the highest performers.

**Figure 2:**
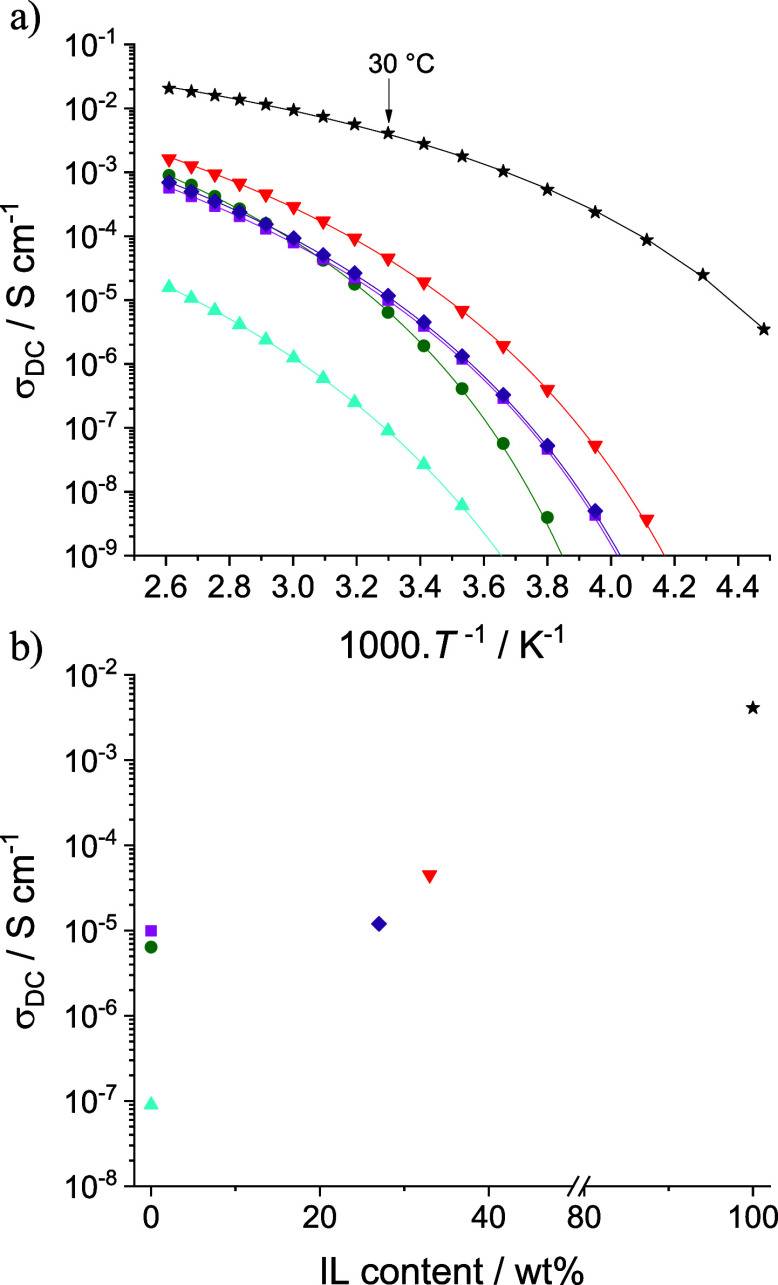
Ionic conductivity, σ_DC_, measured by BDS (a) as a function of reciprocal temperature and (b) at 30 °C depending on the free IL content for PIL^+^ (green circles), PIL^–^ (pink squares), DIG (purple diamonds), DIG_ext_ (blue triangles), DIG_IL_ (red triangles), and pure IL (black stars). The solid lines in (a) represent the best VFT fits obtained using σ_∞_, *B*, and *T*_VFT_ parameters in Table S2.

PIL^+^ and PIL^–^ were then used for the preparation of DIGs through coacervation in acetone (a low boiling common solvent). An acetone solution of PIL^+^ was added dropwise to an acetone solution of PIL^–^, targeting the stoichiometry between ion pairs from each PIL. The formation of a hazy precipitate was immediate, with further vortexing yielding, depending on the solid content, either a single-phase translucent gel-like coacervate (Figure S15 and Movie S1 for 20% wt/vol) or a phase-separated mixture of a translucent elastic solid and a clear liquid (Figure 3 for 3.4% wt/vol). Acetone was evaporated under reduced pressure, and further annealing for 24 h at 70 °C under vacuum yielded a DIG through the formation of dynamic ionic cross-links and concomitant release of IL (Scheme 1). Although it contains 67 wt % of the unentangled PIL^–^, the resulting DIG is a rubbery solid with a macroscopic appearance comparable to that of PIL^+^. The *T*_g_ of DIG (*T*_g_ = −39 °C) lies between those of PIL^+^ and PIL^–^ (*T*_g_ = −30 and −46 °C, respectively), consistent with the value calculated from the Fox equation (*T*_g_ = −40 °C). While this could imply simple mixing behavior between the two polymers, the results presented below provide further evidence that ion exchange between PIL^–^ and PIL^+^ leads to the formation of transient ionic cross-links and IL release. This intermediate value may reflect an increase in *T*_g_ due to the formation of ionic cross-links and a decrease in *T*_g_ due to the plasticization of the ionic network by released IL.

**Figure 3:**
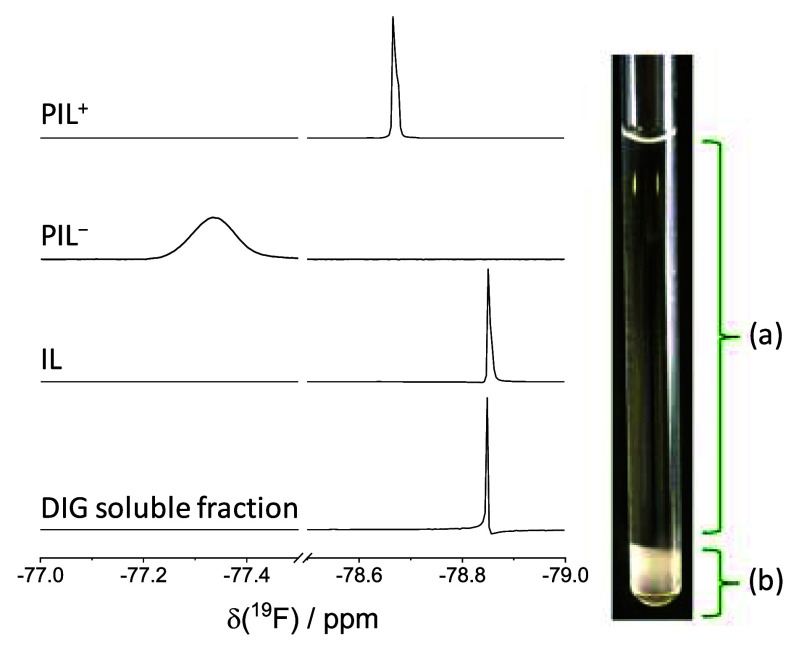
(left) Stack of ^19^F NMR spectra (acetone-*d*_6_) of PIL^+^, PIL^–^, and IL and the soluble fraction of the DIG coacervate; (right) digital image of the NMR tube showing the analysis zone of the DIG soluble fraction (a) and the phase-separated coacervate (b).

The ionic conductivity at 30 °C of the DIG (σ_DC_ = 1.2 × 10^–5^ S cm^–1^) exceeds both that of PIL^–^ (67 wt %, σ_DC_ = 9.9 × 10^–6^ S cm^–1^) and PIL^+^ (33 wt %, σ_DC_ = 6.4 × 10^–6^ S cm^–1^, Figure 2a). DIGs with identical *T*_g_ and σ_DC_ values were obtained when adding PIL^–^ to PIL^+^ instead of PIL^+^ to PIL^–^ during the coacervation step, as well as when targeting polymer weight fractions ranging from 1 to 20% (wt/vol) or when substituting acetone (ε_r_ = 20.7) for methanol (ε_r_ = 32.6) or acetonitrile (ε_r_ = 36.0).^29−35^ Regardless of the physical form of the coacervate (solid-like precipitate, liquid-like, or colloidal) obtained due to variations in the aforementioned conditions, thermal annealing yielded DIGs with identical physical, thermal, and ion conducting properties (results not shown).

The effective formation of ionic cross-links in DIGs was confirmed by quantifying IL release, both by solvent extraction (*ex situ*) and quantitative ^19^F NMR spectroscopy (*in situ*). First, an annealed DIG was extracted with acetone, then subjected to extensive drying under vacuum to yield a coacervate-rich solid fraction (DIG_ext_). The extracted liquid fraction amounted to 24.6 wt % of the initial PIL mixture, and ^1^H NMR spectroscopy (Figure S24) confirmed that it was composed exclusively of the released IL, with no signals characteristic of PIL^+^ or PIL^–^. The amount of extracted IL suggests that ca. 93.9% of ion pairs within PIL^–^ and PIL^+^ have undergone interchain ion exchange.

To confirm that IL release is not a consequence of the extraction process, quantitative ^19^F NMR spectroscopy was also carried out *in situ* on a DIG prepared in an NMR tube by mixing acetone solutions of PIL^+^ and PIL^–^ while targeting a 1:1 stoichiometry between complementary ion pairs and using 1,4-bis(trifluoromethyl)benzene (TFM2B) as internal standard. At such concentration (i.e., 3.4 wt/vol), coacervation led to phase separation and precipitation of the polymer-rich complex coacervate at the bottom of the NMR tube, allowing for analysis of the supernatant solution alone. Comparison of the ^19^F NMR spectra for PIL^+^, PIL^–^, IL, and DIG (Figure 3) corroborates the exclusive presence of IL in the supernatant and the absence of signals corresponding to PIL^+^ or PIL^–^. Integration of the ^19^F signals of the released IL and the TFM2B internal standard (Figure S25) suggests that 90.8% of ion pairs of PIL^+^ and PIL^–^ have undergone interchain ion metathesis, in good agreement with the ex-situ measurement. Besides experimental error, the rather small difference might result from the fact that quantifications by ^19^F NMR and solvent extraction were carried out before and after annealing, respectively.

Comparing further the physical properties, it was found that the *T*_g_ of DIG_ext_ is −30 °C, while the *T*_g_ of DIG is −39 °C (Tables 1 and S3). Additionally, σ_DC_ of DIG_ext_ at 30 °C is more than 2 orders of magnitude lower than of the DIG (σ_DC_ = 9.0 × 10^–8^ and 1.2 × 10^–5^ S cm^–1^, respectively), highlighting that the ionic conductivity is dominated by the released free IL (Figure 2b). To further improve ion conduction, a DIG containing a total IL content of approximately 40 wt % (DIG_IL_) was targeted. The same process was applied (i.e., coacervation in acetone, evaporation, and annealing), but an additional amount of free IL was added in the PIL^–^ solution before coacervation (corresponding to 16.2 wt % in the final material assuming a release of 23.8 wt % of free IL during the formation of ionic cross-links as for DIG). Quantification of the total released IL by *in situ* quantitative ^19^F NMR spectroscopy resulted in only 33 wt % in this case, likely due to displacement of the equilibrium between free ion pairs and ionic cross-links. However, the increase in IL content nonetheless led to a decrease in *T*_g_ for DIG_IL_ to −49 °C due to enhanced plasticization. In parallel, a 4-fold increase in σ_DC_ at 30 °C from 1.2 × 10^–5^ S cm^–1^ for DIG to 4.5 × 10^–5^ S cm^–1^ for DIG_IL_ was observed. While the resultant conductivity is below that of the neat IL (σ_DC_ at 30 °C = 4.1 × 10^–3^ S cm^–1^), it is then significantly higher than that of both the neat PIL^+^ and PIL^–^ (Table 1). Attempts to produce a DIG_IL_ having an IL content of 50 wt % yielded partial exudation of the IL from the DIG, revealing a limitation for this system. Finally, the *T*_onset_ value of 250 °C measured by TGA reveals that the DIG has a thermal stability comparable to PIL^–^ and somehow lower than PIL^+^ and IL (*T*_onset_ = 240, 290, and 320 °C, respectively, Table 1). The limiting thermal stability of DIG is thus related to the ionically cross-linked network (*T*_onset_ = 220 °C for DIG_ext_).

The viscoelastic properties of DIG, DIG_ext_, and DIG_IL_ were measured between −20 and 75 °C and analyzed through TTS referenced at *T*_0_ = *T*_g_ + 40 K (Figure 4). While the DIG has a macroscopic appearance comparable to PIL^+^ (Figure S15), it exhibits specific rheological features distinct from PIL^+^ and PIL^–^ (Figure 1). At high frequencies (10° < ω·*a*_T_ < 10^3^ rad s^–1^), *G*′ and *G*″ of the DIG nearly overlap, with a ω^0.5^ dependence spanning over seven decades of frequency. At lower frequencies (10^–5^ < ω·*a*_T_ < 10° rad s^–1^), the typical disentanglement behavior seen for PIL^–^ is observed, with the exception that the plateau moduli (denoted by asterisks on Figure 4a, *G*_*N*_ = *G*′(ω)_min(tan δ)_ ≈ 80 kPa for DIG) become higher for DIG_ext_ (*G*_*N*_ ≈ 100 kPa) and lower for DIG_IL_ (*G*_*N*_ ≈ 36 kPa). This is consistent with the plasticization and dilution of the entanglements caused by the presence of IL. The ω^0.5^ dependence is typical of a near-critical gel in the vicinity of the sol–gel transition,^36,37^ and indicates that the formation of ion pairs across PILs results in transient cross-links with a large distribution of lifetimes ranging from 1 ms to 1 s at the reference temperature (0 °C). Such near-critical gel behavior was previously observed for aqueous complex coacervates.^37−39^ The significant left-shift of the TTS for lower amounts of IL may not be due to plasticization alone, as the master curves are already referenced at *T*_0_ = *T*_g_ + 40 K. The presence of IL also dramatically decreases the lifetime of transient ionic cross-links, as evidenced by the increase in crossover frequency (denoted by arrows on Figure 4a) by a factor of ca. 60 from DIG_ext_ to DIG_IL_. Although the increased amount of IL in DIG_IL_ enhanced the ion conducting properties compared to DIG (σ_DC_ = 3.0 × 10^–5^ and 7.2 × 10^–6^ S cm^–1^ at 25 °C, respectively), it also decreased stiffness (*G*′ for DIG_IL_ at 25 °C and 1 rad s^–1^ ∼ 4 kPa is six times lower than for DIG, Figure 4c).

**Figure 4:**
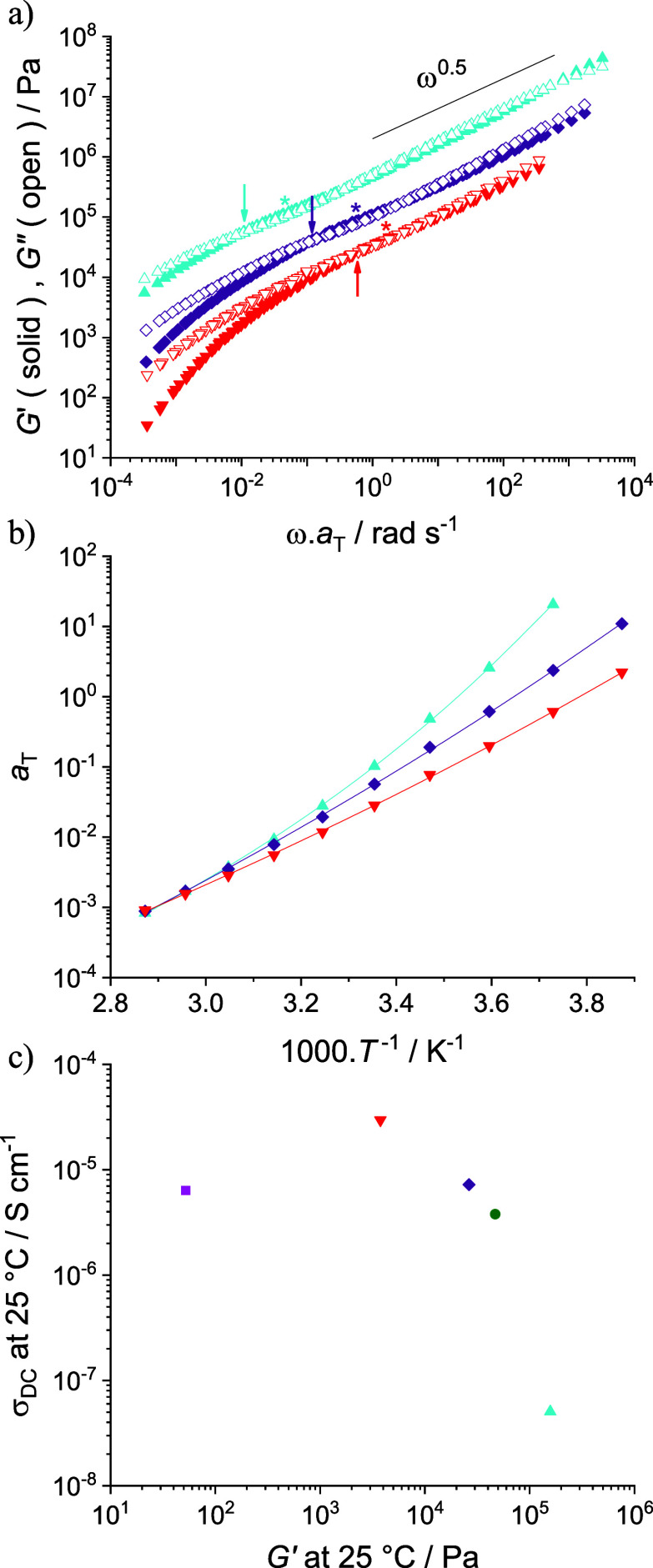
(a) TTS master curves referenced at *T*_0_ = *T*_g_ + 40 K for DIG_ext_ (blue triangles), DIG (purple diamonds), and DIG_IL_ (red triangles) obtained by SAOS rheology at temperatures ranging from −20 to 75 °C. (b) Corresponding *a*_T_ shift factors and WLF best fits at *T*_0_ = *T*_g_ + 40 K for DIG_ext_ (blue triangles), DIG (purple diamonds), and DIG_IL_ (red triangles). (c) Ionic conductivity, σ_DC_, measured by BDS at 25 °C as a function of the storage modulus (*G*′) obtained at 25 °C and 1 rad s^–1^ for PIL^+^ (green circle), PIL^–^ (pink square), DIG_ext_ (blue triangle), DIG (purple diamond), and DIG_IL_ (red triangle). The arrows represent crossover frequencies, and the asterisks represent plateau moduli taken as the minimum of tan(δ).

We have demonstrated an innovative, versatile, yet straightforward method for the preparation of quasi-solid polymer electrolytes by coacervation of oppositely charged PILs through ion metathesis, generating ionic cross-links and concomitant release of IL. Although coacervation is sensitive to experimental conditions (solvent, order of mixing, dilution, temperature, etc.), solvent evaporation, and annealing consistently produced equivalent DIGs, independent of the initial state of the coacervate. The formation of ionic cross-links through ion metathesis and the beneficial impact of released IL was demonstrated by DSC and BDS. Although the interchain cross-linking of complementary charged PILs could be demonstrated from rheology, such cross-links are transient and feature fast exchange dynamics–further accelerated by the presence of released IL. It appears that the anhydrous ionic conductivity of the DIGs can be significantly improved by the addition of IL (σ_DC_ at 30 °C increases from 9.0 × 10^–8^ to 4.5 × 10^–5^ S cm^–1^ when the IL content increases from 0 to ∼33 wt %). However, the viscoelastic properties are strongly affected (*G*′ at 25 °C and 1 rad s^–1^ decreases from 158 to 4 kPa when IL content increases from 0 to ∼33 wt %) due to plasticization and the dilution of entanglements in the presence of IL. Finally, while this system involves analogous ion pairs in the polycationic and polyanionic precursors, we are confident that DIG properties could be further tailored and improved by further structural design of the building blocks (e.g., chemical nature and polymerization degree of the PILs, content and chemical nature of the ion pairs, extension to block copolymers including rigid segments able to self-assemble and enhance mechanical properties).

## Supporting Information Available

Full synthetic and characterization details, including ^1^H, ^13^C, and ^19^F NMR, FTIR, DSC, TGA, and BDS data (PDF)
Movie S1 (MP4)
