# Supplementary material for: Dynamic Ion Gels from the Complex Coacervation of Oppositely Charged Poly(ionic liquid)s
Source: ACS Macro Lett. 2024 Jul 11;13(8):921–7. doi: 10.1021/acsmacrolett.4c00253 (PMC11340024; doi:10.1021/acsmacrolett.4c00253)
Supplement: Supplementary file 1 — mz4c00253_si_001.pdf [file mz4c00253_si_001.pdf]

Supporting Information for

Dynamic Ion Gels from the Complex Coacervation of Oppositely Charged Poly(ionic liquid)s.

Malak Alaa Eddine,[†,a] Daniil R. Nosov,[†,b,c] Luiz Fernando Lepre,[a] Anatoli Serghei,[a] Daniel F. Schmidt,[b] Damien Montarnal,[d] Alexander S. Shaplov,[*b] and Eric Drockenmuller[*a]

[a)] *Université Claude Bernard Lyon 1, CNRS, Ingénierie des Matériaux Polymères, UMR 5223, Lyon, F-69003, France*
[b)] *Luxembourg Institute of Science and Technology (LIST), 5 avenue des Hauts-Fourneaux, L-4362 Esch-sur-Alzette, Luxembourg*
[c)] *Department of Physics and Materials Science, University of Luxembourg, 2 Avenue de l'Université, L-4365 Esch-sur-Alzette, Luxembourg*
[d)] *Université Claude Bernard Lyon 1, CPE Lyon, CNRS, Catalyse, Polymérisation, Procédés et Matériaux, UMR 5128, Lyon, F-69003, France*

[†] *Contributed equally*

Corresponding Authors: *****E-mail: alexander.shaplov@list.lu

*****E-mail: eric.drockenmuller@univ-lyon1.fr

TABLE OF CONTENTS

I. Materials S3

II. Characterization methods

Nuclear magnetic resonance (NMR) S3

Infrared spectroscopy (IR) S4

Size exclusion chromatographie (SEC) S4

Differential scanning calorimetry (DSC) S5

Thermogravimetric analysis (TGA) S5

Rheological measurements S5

Broadband dielectric spectroscopy (BDS) S6

III. Synthesis of poly(ionic liquid)s

Synthesis of polycation PIL^+ S7

Synthesis of ILM S13

Synthesis of polyanion PIL^- S16

IV. Preparation of dynamic ion gels (DIGs)

General procedure for the preparation of DIGs S21

V. Determination of the amount of released ionic liquid

Gravimetry (preparation of DIG_{ext}) S27

Quantitative ^{19}F NMR spectroscopy S28

Preparation of IL-doped DIG (DIG_{IL}) S29

VI. Tables S30

VII. References S34

I. Materials

Acetone (99.8%, Carlo Erba), dichloromethane (DCM, 99.6%, Acros), ethyl acetate (99.6%, Acros), acetonitrile (99.5%, Merck), methanol (99.9%, Carlo Erba), *N*,*N*-dimethylformamide (DMF, anhydrous 99,8%, Acros), 1-butyl-3-methylimidazolium bromide (99%, Iolitec), magnesium sulfate ($MgSO_4$, 99.5%, Merck), 4-methoxyphenol (99%, Merck), poly(ethylene glycol) methyl ether methacrylate (PEGM, M_n ~ 500 g mol^{-1}, Merck), 4-cyano-4-(phenylcarbonothioylthio)pentanoic acid (CPCP, Merck), lithium bis(trifluoromethylsulfonyl)imide (LiTFSI, 99%, Solvionic), poly(epichlorohydrin-*s*-ethylene oxide) (Hydrin C2000, m:n = 1:1, Zeon Europe GmbH) were used as received. Azobisisobutyronitrile (AIBN, Sigma Aldrich) was recrystallized from methanol. *N*-Butylimidazole was distilled under inert atmosphere over CaH_2. Lithium 1-[3-(methacryloyloxy)propylsulfonyl]-1-(trifluoromethanesulfonyl)imide was synthesized following a three step procedure in full accordance with previous reports.[1-2] 1-Butyl-3-methylimidazolium bis(trifluoromethylsulfonyl)imide ($[C_4C_1Im][TFSI]$, IL, 99.5%, Solvionic) was dried under vacuum for 48 h at 80 °C.

II. Characterization methods

Nuclear magnetic resonance. NMR spectra were recorded on AMX-600 and Bruker Avance 500 spectrometers (Bruker, Germany) at 25 °C in the indicated deuterated solvents and are listed in part per million (δ, ppm). The structure of synthesized ILM and PILs were verified on AMX-600 spectrometer at 25 °C in DMSO-d_6. The signals corresponding to the residual protons (^{1}H: δ = 2.50 ppm) and carbons (^{13}C: δ = 39.52 ppm) of the DMSO-d_6 were used as internal standards for ^{1}H and ^{13}C NMR, respectively. The C_6F_6 was added to the solutions in deuterated solvent and was utilized as an external standard (^{19}F: δ = -164.9 ppm) for ^{19}F NMR.

The characterization of PILs, DIGs and IL release was done by recording of ^{1}H and ^{19}F NMR spectra on a Bruker Avance 500 spectrometer in acetone-d_6 at 25 °C using a Bruker BBFO ^{1}H / ^{109}Ag-^{19}F 5mm gradient Z probe. Chemical shifts are reported relative to the acetone-d_6 residual proton peaks (^{1}H: δ = 2.05 ppm) for ^{1}H NMR or relative to an external reference for ^{19}F NMR (^{19}F: δ = 0.00 ppm, $CFCl_3$). Abbreviations for peak multiplicity are given as follows: s for singlet, d for doublet, t for triplet, q for quartet, p for pentet, dt for doublet of triplets, sext for sextet, m for multiplet and br.s for broad.

IR spectra were acquired on a Brucker Tensor 27 Fourier IR-spectrometer using ATR technology (128 scans, 2 cm^{-1} resolution) and Spectragryph optical spectroscopy software.[3]

Size exclusion chromatography (SEC). A 1200 Infinity gel permeation – size exclusion chromatograph (SEC, Agilent Technologies) was used to determine number average molar masses (M_n), weight average molar masses (M_w) and chain dispersities ($Đ = M_w/M_n$) of PILs. The chromatograph was equipped with an integrated IR detector, 0.1 M solution of $Li(CF_3SO_2)_2N$ in DMF was used as an eluent at 50 °C and the flow rate was set as 1.0 mL min^{-1}. A set of PL PolarGel-M column and a PL PolarGel-M guard column (Agilent Technologies) was applied for analysis of anionic PIL^{-}, while another set of TSKgel G5000-H_{HR} column and TSKgel H_{HR}-H guard column (Tosoh Bioscience) was used to study cationic PIL^{+}. Polymer solutions (4 mg mL^{-1}) in 0.1 M solution of $Li(CF_3SO_2)_2N$ in DMF were filtered through 0.20 µm pore size polytetrafluoroethylene (PTFE) filters prior to the measurements. Polymethylmethacrylate standards (EasiVial PMMA, Agilent Technologies, M_p = 550–1568000 g mol^{-1}) were used to perform calibration.

Differential scanning calorimetry (DSC) measurements were done using a DSC Q200 (TA Instrument) calibrated with an indium standard. The samples were prepared in aluminium hermetic pans and the experiments were conducted under a nitrogen purge of 25 mL min^{-1} on *ca*. 5-10 mg samples. The sample was first heated to 100 °C at a rate of 10 °C min^{-1} and isothermally annealed for 1 min. Then, the temperature was decreased to –70 °C at a rate of 10 °C min^{-1} followed by a second heating to 100 °C at a rate of 10 °C min^{-1}. The glass transition temperatures (T_g) were measured at the mid-point of the transition (on the second heating cycle) using the TA Thermal Analysis software.

Thermogravimetric analysis (TGA) was performed using a TGA Q500 (TA Instruments). A heating ramp from 30 to 700 °C was applied at 10 °C min^{-1} under a helium purge of 60 mL min^{-1} to *ca*. 5-15 mg samples. The onset weight loss temperature (T_{onset}) was determined as the point in the TGA curve at which a significant deviation from the horizontal was observed. The resulting temperature was then rounded to the nearest 5°C.

Rheological measurements were performed using a strain-controlled ARES-G2 rheometer (TA Instruments) equipped with an ACS-3 chiller (TA Instruments). Plate-plate disposable aluminum geometries with a diameter of 25 mm were adopted for neat and previously dried PILs. In the case of the dried DIGs, smaller disposable aluminum plates with a diameter of 8 mm were used. Aiming to eliminate the effect of moisture on the viscoelastic properties of the ionic samples, both PILs and DIGs were dried inside a vacuum oven for at least 24 h at 70 °C before the measurements. To guarantee a maximum contact between the sample and the geometry, a sufficiently thick layer of 0.4–1.5 mm and an initial positive axial force was applied. Frequency sweep tests were conducted at temperatures ranging from -20 °C to 75 °C from 628 rad s^{-1} to 0.1 rad s^{-1}. To determine the linear viscoelastic region of each sample preliminary strain sweep tests were carried out at a fixed

frequency of 1 rad s^{-1} in a range of deformation between 0.01 and 100%. The master curves were built through time-temperature superposition and were referenced at $T_0 = T_g + 40$ K. The corresponding shift factors a_T were determined and it was verified that they present a temperature dependence that follows the WLF (Williams-Landel-Ferry) **equation S1**[4]:

$$\log(a_T) = -\frac{C_1(T-T_0)}{C_2+(T-T_0)} \quad \text{(eq S1)}$$

The empirical parameters C_1 and C_2 obtained for $T_0 = T_g + 40$ K are listed in **Table S1**.

Broadband dielectric spectroscopy (BDS) was employed to measure the effect of temperature on the ionic conductivity of the samples. The measurements were carried out using a high resolution Alpha–Analyzer (Novocontrol GmbH). The sample temperature was controlled under a flow of pure nitrogen gas (Quatro temperature controller) so that any presence of oxygen and moisture in the measuring chamber can be excluded. The thermal stability was better than 0.1 K, with relative variations less than 0.2 K min^{-1}. For PIL^+ and PIL^-, a solution of 100 mg in acetone (1.5 mL) was deposited drop wise onto a platinum electrode (2 cm in diameter) and the solvent was slowly evaporated under ambient conditions. For DIG, DIG_{ext} and DIG_{IL}, ca. 100 mg of previously dried samples at 70 °C under vacuum for at least 24 h were directly placed in the platinum electrode with a stainless steel spatula. In order to remove any traces of solvent or water in the samples, a thermal annealing at 70 °C under vacuum for 18 h was carried-out. After the thermal treatment, a second platinum electrode (3 cm in diameter) was placed on top of the sample to build up a measurement cell as a parallel plate capacitor. The sample thickness was controlled by employing 100 μm thick Teflon spacers. A further annealing was performed inside the cryostat of the dielectric spectrometer under a flow of pure nitrogen during 3 h at 110 °C. The electrical and dielectric properties were continuously monitored until the equilibrium was reached. Once this equilibration procedure was

done, the ionic conductivity measurements were started by measuring the complex conductivity function which is defined by **equation S2**:

$$\sigma^*(\omega,T) = \sigma'(\omega,T) + i\sigma''(\omega,T) \quad \text{(eq S2)}$$

The conductivity measurements were carried-out from 10 MHz to 0.1 Hz under isothermal frequency sweeps from 110 to −50 °C in temperature steps of 10 °C The applied voltage was 0.1 V to exclude any possible non–linear effects. Once the measurements were finished the samples were re-measured at a reference temperature chosen as 30 °C to validate that the experimental results were reproducible and stable. The DC-conductivity values (σ_{DC}) of the anhydrous PILs and DIGs were obtained by the value of the plateau observed in the frequency dependence of σ′ (**Figure S18-S22**). Above T_g, the dependence of σ_{DC} on the inverse temperature follows a typical Vogel–Fulcher–Tammann (VFT) behavior for all studied samples. The experimental results were thus fitted with the VFT **equation S3**:

$$\sigma_{DC} = \sigma_\infty \times exp\left(-\frac{B}{(T-T_{VFT})}\right) \quad \text{(eq S3)}$$

with σ_{DC} the ionic conductivity in the high temperature limit, B a fitting parameter related to the activation energy of the ionic conduction, and T_{VFT} the Vogel temperature. The obtained parameters are listed in **Table S2**.

III. Synthesis of poly(ionic liquid)s

Synthesis of poly(1-butyl-3-[oxiran-2-ylmethyl]-1-imidazol-3-ium-*s*-ethylene oxide) bis(trifluoromethylsulfonyl) imide (PIL$^+$). PIL$^+$ was synthesized in two steps involving quaternization of *N*-butylimidazole by poly(epichlorohydrin-*s*-ethylene oxide) and ion metathesis of the obtained chloride polyelectrolyte with an excess of $Li(CF_3SO_2)_2N$ (**Scheme S1**).

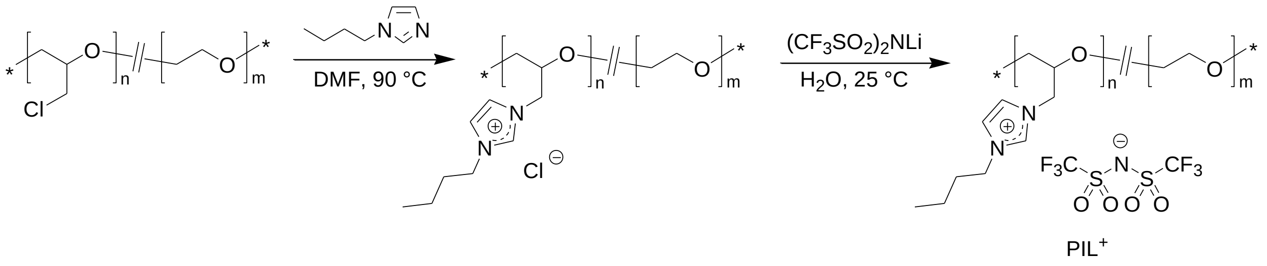

Scheme S1. Synthesis of polyether/imidazolium/TFSI-based cationic PIL^+.

Poly(epichlorohydrin-*s*-ethylene oxide) Hydrin C2000 (3.27 g, 24.0 mmol of chloromethyl groups) was dissolved in 40 mL of anhydrous DMF under inert atmosphere at 70 °C in a heavy wall glass pressure vessel. *N*-Butylimidazole (29.8 g, 240 mmol) was added in one portion to the polymer solution. The temperature was raised to 90 °C and stirring was continued for 3 days. The solution was cooled down and ~1/2 of the solvent was removed under reduced pressure at 65 °C. Afterwards polymer was precipitated into 200 ml of acetone. Poly(1-butyl-3-[oxiran-2-ylmethyl]-1-imidazol-3-ium-*co*-ethylene oxide) chloride as sticky light brown mass was thoroughly washed with acetone and dried at 70 °C/0.1 mbar for 1 day. Yield: 4.50 g (72.1%). ^{1}H NMR (600 MHz, D_2O) δ(ppm): 7.59-7.56 (m, 2H), 4.57-4.33 (m, 2H), 4.25 (br.s., 2H), 4.01 (br.s., 1H), 3.93-3.53 (m, 8H) 1.88 (br.s., 2H), 1.32 (br.s., 2H), 0.94 (br.s., 3H). Quaternization degree (**Q**) ≈ 90%.

The solution of lithium bis(trifluoromethylsulfonyl)imide (9.00 g, 34.6 mmol) in 30 mL of milli-Q water was added dropwise to the aqueous solution (100 ml) of poly(1-butyl-3-[oxiran-2-ylmethyl]-1-imidazol-3-ium-*r*-ethylene oxide) chloride (4.50 g, 17.3 mmol) at room temperature. The formation of a suspension was immediately observed and the stirring was continued for 2 h at RT. The precipitated sticky product was collected, washed with water, redissolved in acetone and precipitated in water again. Polymer representing transparent slightly yellow sticky mass was thoroughly washed with H_2O, dried at 70 °C /0.1 mbar for 2 days in B-585 oven (Buchi Glass Drying Oven, Switzerland) filled with P_2O_5. Yield: 6.62 g (76.2%). M_n = 110000 g mol^{-1}, M_w/M_n = 3.45 (by GPC); T_g = -30 °C (by DSC); T_{onset} = 290°C (by TGA in air); $\sigma_{DC} = 6.4 \times 10^{-6}$ Sm cm$^-$

[1] (30 °C); ^{1}H NMR (600.2 MHz, DMSO-d_6) δ(ppm): 9.06 (s, 1H), 7.80 (s, 1H), 7.65 (s, 1H), 4.38-4.19 (m, 4H), 3.85-3.51 (m, 7H), 1.79 (s, 2H), 1.25 (s, 2H), 0.90 (s, 3H) (for the detailed assignment see **Figure S1**); ^{13}C NMR (150.9 MHz, DMSO-d_6) δ(ppm): 136.6, 123.4, 122.10, 119.5 (q, J_{CF} = 322.3 Hz), 77.5, 75.9, 70.1, 69.7, 68.7, 49.8, 48.6, 44.0, 31.4, 18.7, 13.2 (**Figure S2**); ^{19}F NMR (564.7 MHz, DMSO-d_6) δ(ppm): -80.9 (s) (**Figure S3**); IR (ATR-mode): 3152 (m), 3113 (w), 2966 (m, ν_{CH}), 2939 (m, ν_{CH}), 2879 (m, ν_{CH}), 1564 (m), 1464 (m, ν_{CH}), 1450 (m), 1347 (S, ν_{CH2}), 1329 (s, ν_{asSO2}), 1226 (m, ν_{CF}), 1179 (vs, ν_{sSO2}), 1132 (vs), 1052 (vs, ν_{CF}), 950 (w, ν_{CH2}), 843 (m, $\nu_{CO/CH2}$), 789 (m), 762 (m), 739 (s), 644 (m), 612 (s), 599 (s), 589 (vs), 506 (s) cm^{-1} (**Figure S4**); Calc. for 0.1 $C_5H_9ClO_2$ × 0.9 $C_{14}H_{21}F_6N_3O_6S_2$ (468.16): C, 33.58%; H, 4.26%; Found: C, 33.06%; H, 3.93%.

Determination of the quaternization degree (Q).

The quaternization degree **Q** was determined by ^{1}H NMR using integral in the region of 4.10-3.40 ppm and the following equations:

$$Q = \frac{I^{n}_{PIL^+}}{I^{n}_{PIL^+} + I^{n}_{Hydrin}} \times 100\% \quad \text{(eq S4)}$$

$$I^{n}_{PIL^+} = \frac{I^{theor}_{PIL^+}}{n_{PIL^+}} = \frac{7}{7} = 1 \quad \text{(eq S5)}$$

$$I^{n}_{Hydrin} = \frac{I_{Hydrin}}{n_{Hydrin}} = \frac{I^{exp}_{PIL^+} - I^{theor}_{PIL^+}}{n_{Hydrin}} = \frac{7.81 - 7.0}{9} = 0.09 \quad \text{(eq S6)}$$

$$Q = \frac{1}{1 + 0.09} \times 100\% = 92\% \quad \text{(eq S7)}$$

, where $I^{n}_{PIL^+}$ is the normalized integral for quaternized polymer PIL^+ in the region of 4.10-3.40 ppm, I^{n}_{Hydrin} is the normalized integral for unmodified Hydrin C2000 in the region of 4.10-3.40 ppm, $I^{theor}_{PIL^+}$ is the theoretical integral for fully quaternized polymer PIL^+ in the region of 4.10-3.40 ppm, n_{PIL^+} is the theoretical number of protons corresponding to fully quaternized polymer PIL^+ in region of 4.10-3.40 ppm (**Figure S1b**, signals 6-9), I_{Hydrin} is the integral for unmodified Hydrin C2000 in the region of 4.10-3.40 ppm, n_{Hydrin} is the theoretical number of protons corresponding

to unmodified Hydrin C2000 in region of 4.10-3.40 ppm (**Figure S1a**, signals 1-5), $I_{PIL^+}^{exp}$ is the experimentally determined integral for fully quaternized polymer PIL^+ in the region of 4.10-3.40 ppm.

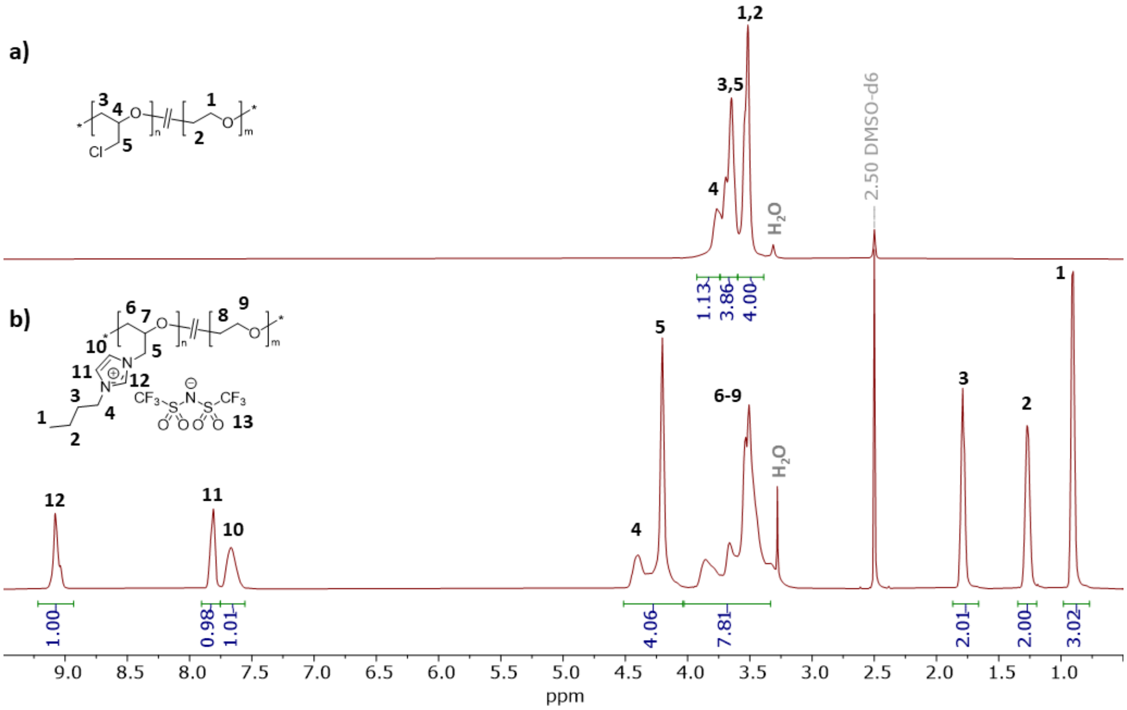

Figure S1. Comparison of ^{1}H NMR of poly(epichlorohydrin-co-ethylene oxide) and polyether/imidazolium/TFSI-based cationic PIL^+ (DMSO-d_6, 25 °C).

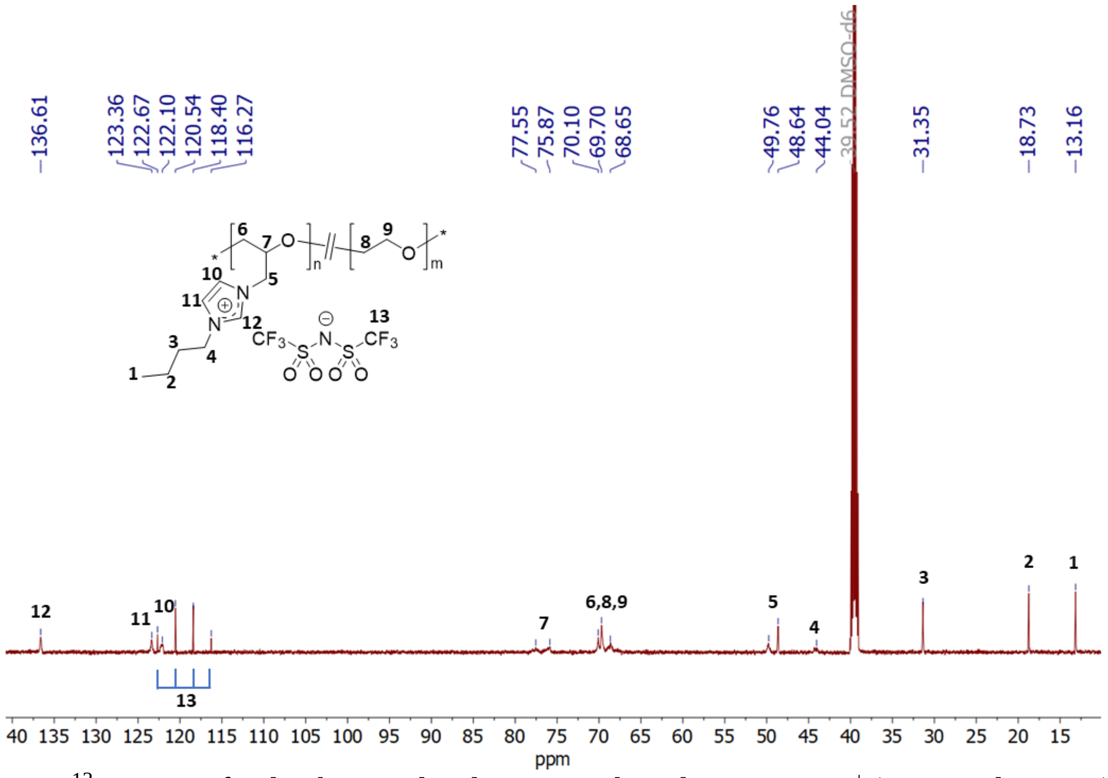

Figure S2. ^{13}C NMR of polyether/imidazolium/TFSI-based cationic PIL^{+} (DMSO-d_6, 25 °C).

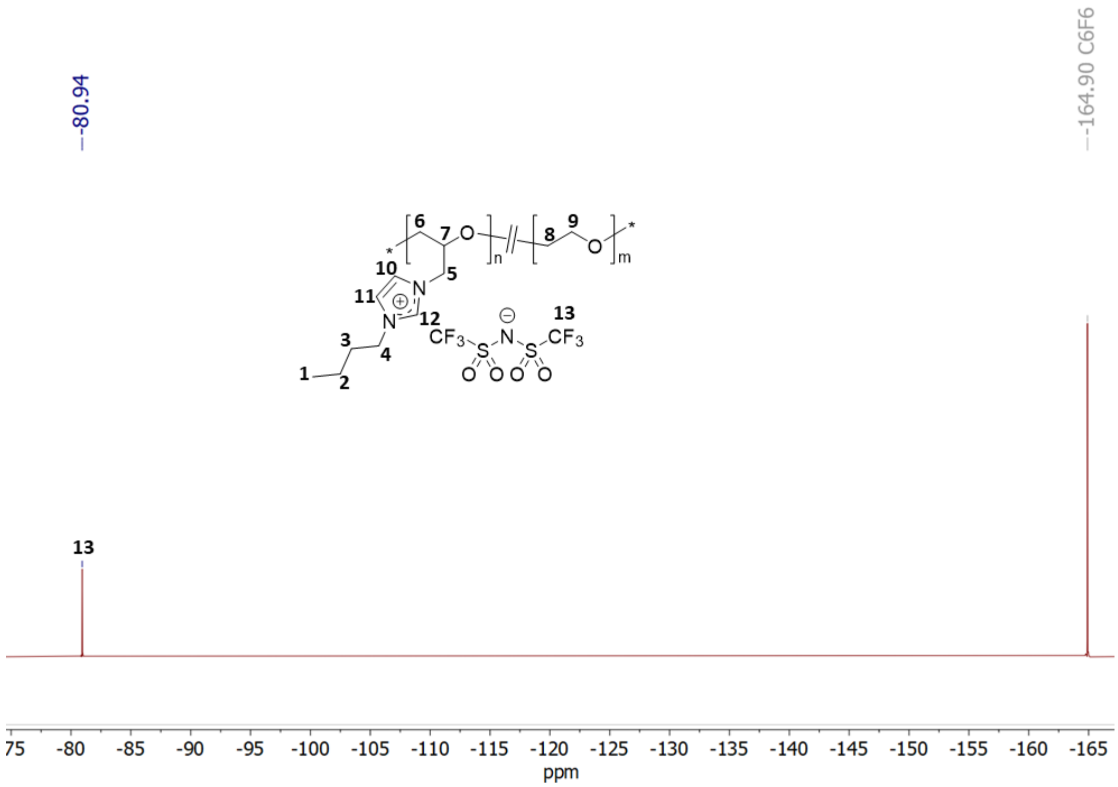

Figure S3. ^{19}F NMR of polyether/imidazolium/TFSI-based cationic PIL^{+} (DMSO-d_6, 25 °C).

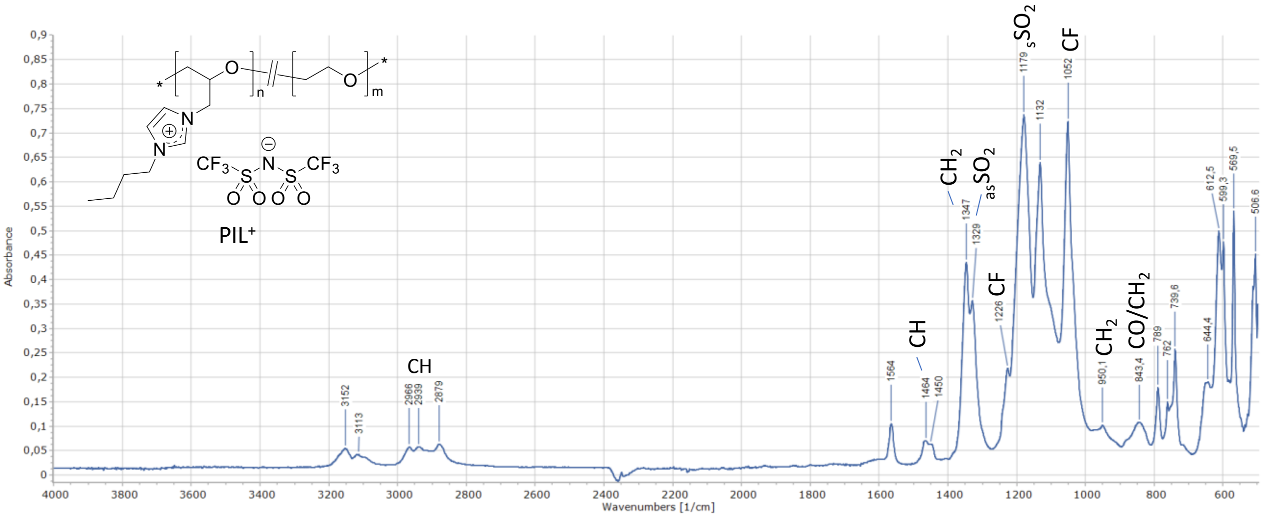

Figure S4. FTIR spectrum of polyether/imidazolium/TFSI-based cationic PIL$^+$.

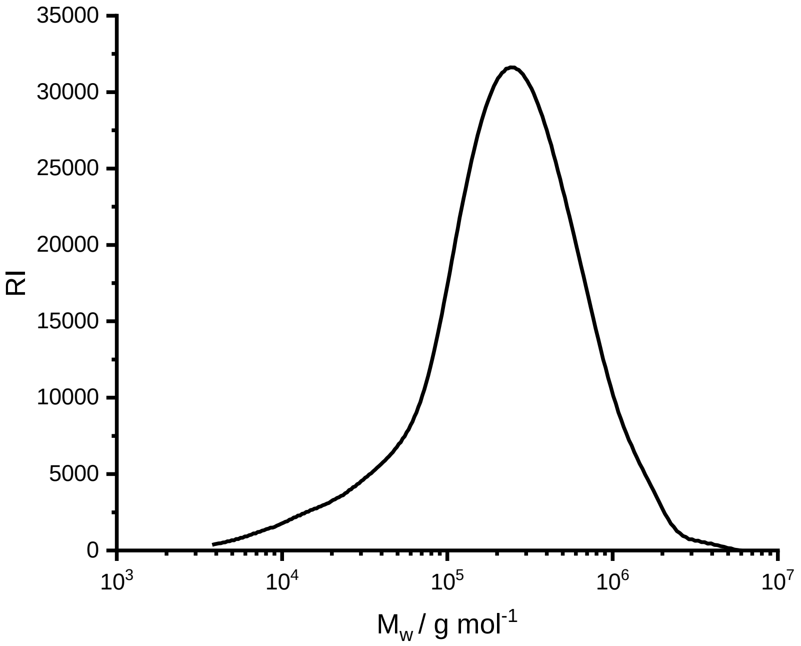

Figure S5. SEC traces (50 °C, 0.1 M LiTFSI in DMF, calibration with PMMA standards) of polyether/imidazolium/TFSI-based cationic PIL$^+$.

Synthesis of 1-butyl-3-methylimidazolium 1-[3-(methacryloyloxy)propylsulfonyl]-1-(trifluoromethanesulfonyl)imide (ILM). This monomer was synthesized from lithium 1-[3-(methacryloyloxy)propylsulfonyl]-1-(trifluoromethanesulfonyl)imide[1-2] via ion metathesis reaction with an excess of 1-metyl-3-butylimidazolium bromide in the aqueous medium (**Scheme S2**).

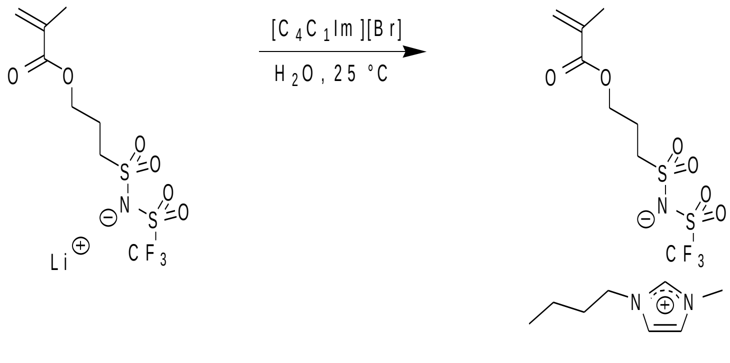

Scheme S2. Synthesis of methacrylate/TFSI/imidazolium-based anionic ILM.

The solution of lithium 1-[3-(methacryloyloxy)propylsulfonyl]-1-(trifluoromethanesulfonyl)imide (32.5 g, 94.0 mmol) in 150 ml of milli-Q water was added dropwise to the aqueous solution (60 mL) of 1-metyl-3-butylimidazolium bromide (21.1 g, 96.2 mmol) at room temperature. The formation of an emulsion was observed immediately and the stirring was continued for 2 h at RT. The organic oil was extracted from reaction mixture with DCM. The DCM layer was washed with water (4 × 30 mL), dried over anhydrous magnesium sulfate. $MgSO_4$ was filtered off, a catalytic amount of 4-methoxyphenol (as an inhibitor) was added and dichloromethane was removed under reduced pressure at RT. Resultant light yellow transparent oil was dried at 25 °C/0.1 mbar for 8 h. Yield: 27.4 g (77.4 %). T_g = -63 °C (by DSC); ^{1}H NMR (600 MHz, DMSO-d_6) δ(ppm): 9.09 (s, 1H), 7.76 (s, 1H), 7.69 (s, 1H), 6.03 (s, 1H), 5.67 (s, 1H), 4.17 (dt, J = 13.4, 6.8 Hz, 4H), 3.85 (s, 3H), 3.10 – 3.04 (m, 2H), 2.06–1.97 (m, 2H), 1.88 (s, 3H), 1.79–1.74 (m, 2H), 1.29–1.23 (m, 2H), 0.90 (t, J = 7.4 Hz, 3H) (for the detailed assignment see **Figure S6**); ^{13}C NMR (150 MHz, DMSO-

d_6) δ(ppm): 166.4, 136.5, 135.8, 125.6, 123.6, 122.2, 120.1 (q, J_{CF} = 324.5 Hz), 62.8, 51.3, 48.5, 35.7, 31.3, 23.5, 18.7, 17.9, 13.1 (**Figure S7**); ^{19}F NMR (564.7 MHz, DMSO-d_6) δ(ppm): -79.8 (s) (**Figure S8**); IR (ATR-mode): 3151 (m), 3114 (m), 2963 (m, ν_{CH}), 2938 (m, ν_{CH}), 2877 (w, ν_{CH}), 1716 (s, $\nu_{C=O}$), 1637 (m, $\nu_{C=C}$), 1572 (m), 1466 (m, ν_{CH}), 1410 (w), 1321(vs, ν_{asSO2}), 1298 (s), 1222 (m, ν_{CF}), 1178 (vs, ν_{sSO2}), 1123 (s), 1052 (s, ν_{CF}), 1023 (m), 945 9w), 817 (m), 754 (m), 712 (w), 649 (m), 622 (s) cm^{-1} (**Figure S9**); Calc. for $C_{16}H_{26}F_3N_3O_6S_2$ (477.51): C, 40.25%; H, 5.49%; N, 8.80%; Found: C, 40.11%; H, 5.40%; N, 8.92%.

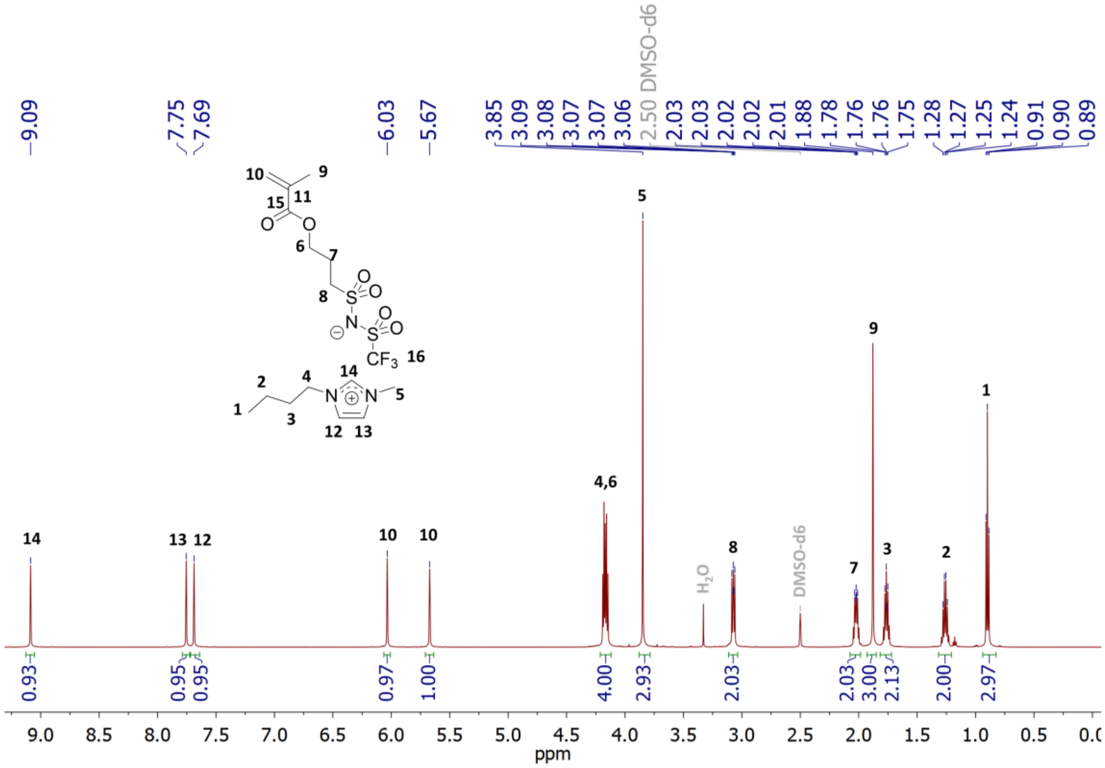

Figure S6. ^{1}H NMR of ILM (DMSO-d_6, 25 °C).

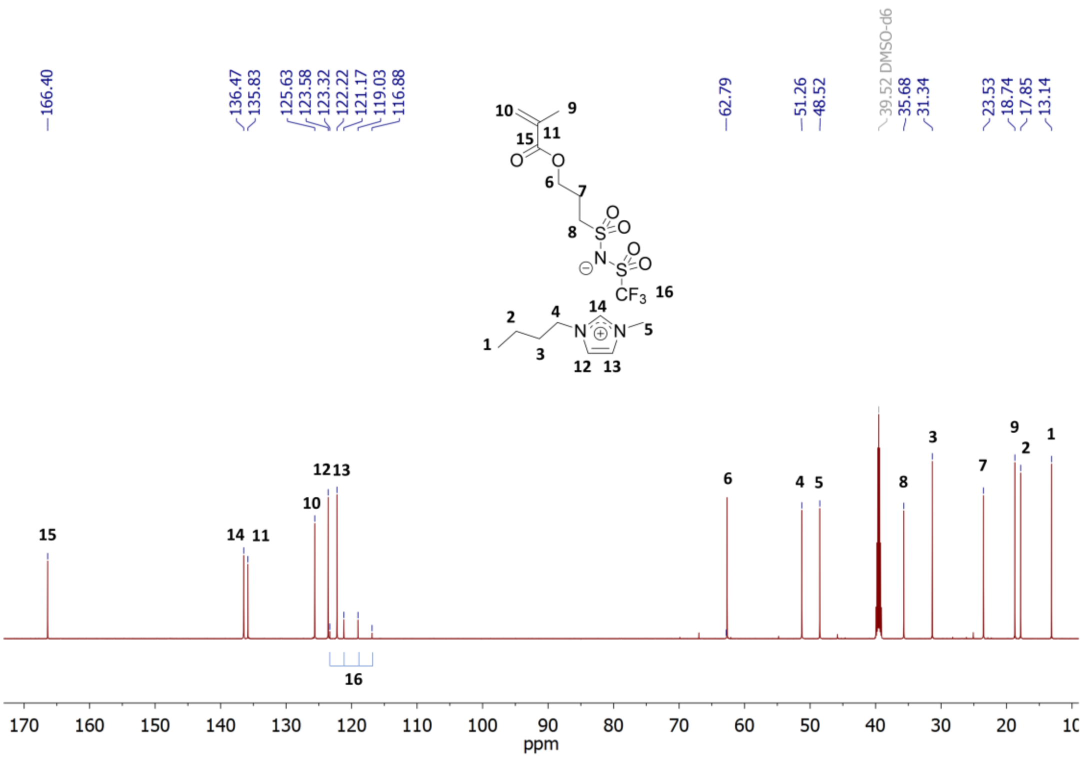

Figure S7. ^{13}C NMR of ILM (DMSO-d_6, 25 °C).

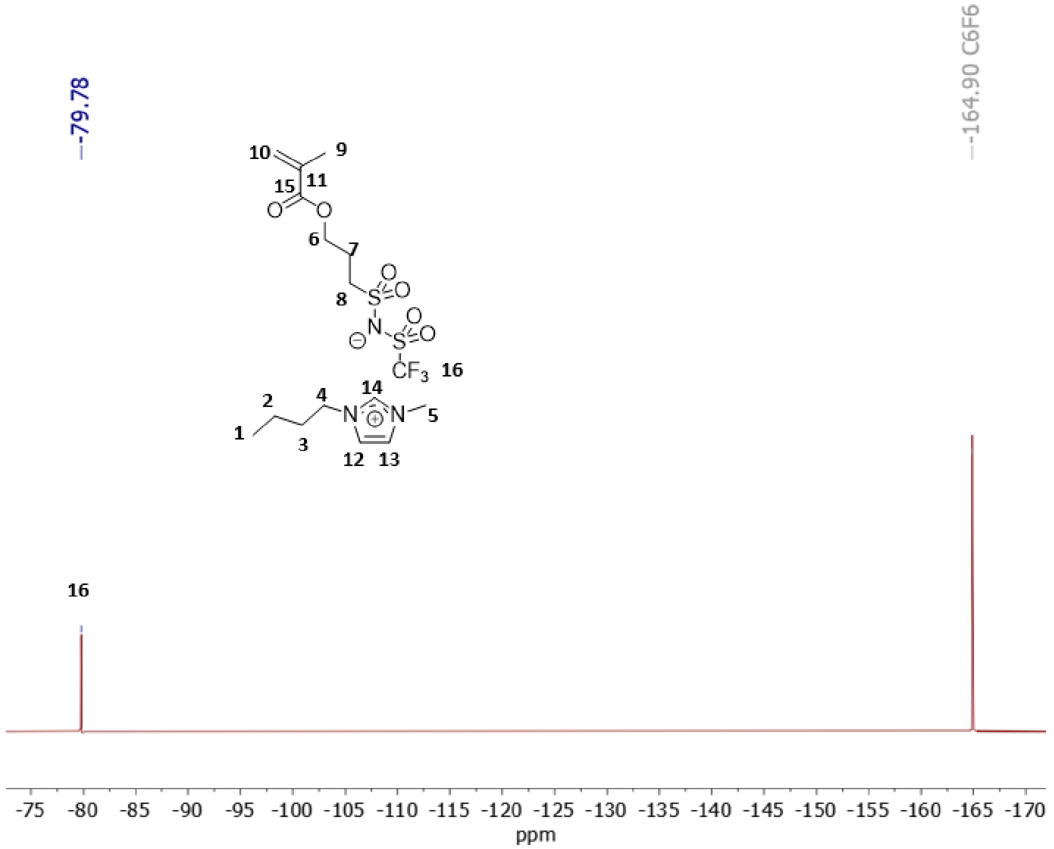

Figure S8. ^{19}F NMR of ILM (DMSO-d_6, 25 °C).

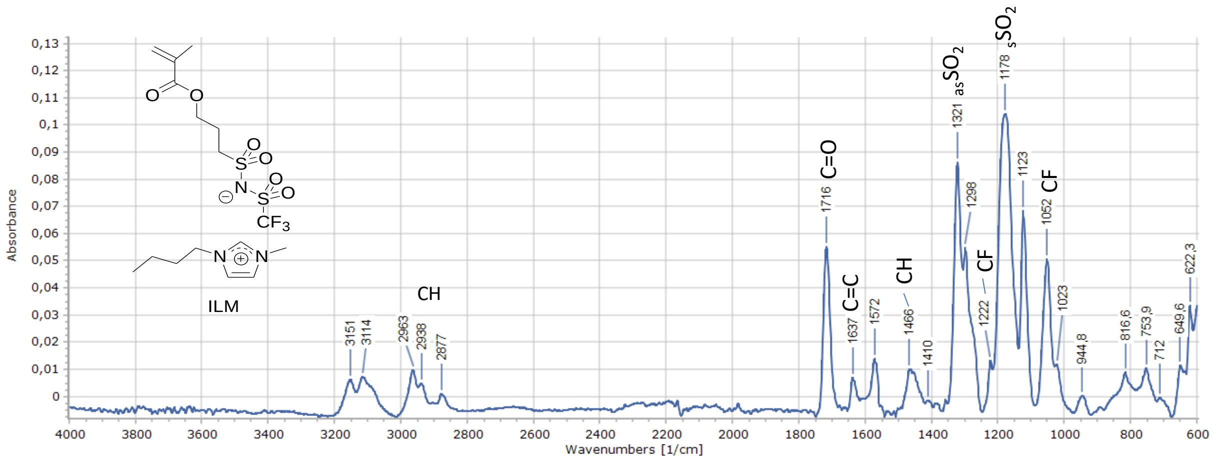

Figure S9. FTIR spectrum of ILM.

Synthesis of poly[(1-butyl-3-methylimidazolium 1-[3-(methacryloyloxy)propylsulfonyl]-1-(trifluoromethanesulfonyl)imide)-*r*-(poly(ethyleneglycol) methyl ether methacrylate)] (PIL⁻). PIL⁻ was prepared via RAFT random copolymerization of ILM and poly(ethylene glycol)methyl ether methacrylate (PEGM) in 1:1 molar ratio. Typical polymerization procedure is given bellow (**Scheme S3**).

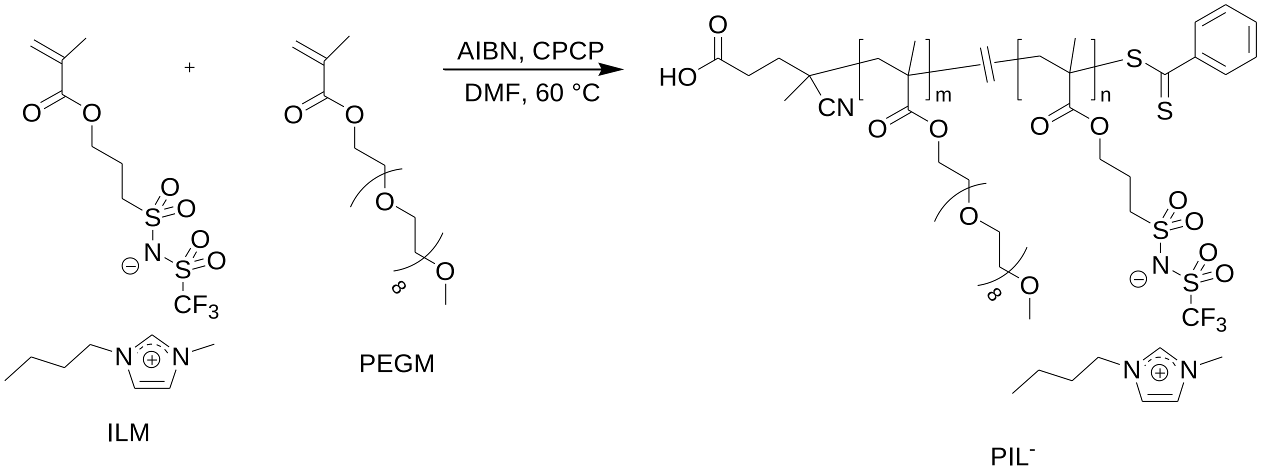

Scheme S3. Synthesis of polymethacrylate/imidazolium/TFSI-based anionic PIL⁻.

A solution of ILM (3.00 g, 6.28 mmol), PEGM (3.14 g, 6.29 mmol), CPCP (17.1 mg, 0.061 mmol) and AIBN (2.01 mg, 0.012 mmol, [AIBN]:[CPCP]=1:5 by mol) in anhydrous DMF (19.5 mL, 18.4 g, [DMF]:[ILM+PEGM]=3:1 by weight) was transferred to a Schlenk flask equipped with magnetic stirring bar. The solution was de-gassed via three freeze-pump-thaw cycles and flashed with argon, whereupon the flask was placed into a bath preheated at 60 °C. Polymerization was further carried out under at 60 °C for 72 h. The resultant viscous pink polymer solution was diluted with DMF and precipitated into the excess of ethyl acetate, PIL^{-} was collected by decantation of the solvents, then redissolved in acetone and precipitated in the excess of ethyl acetate for the second time. Isolated copolymer represented pink sticky mass that was dried at 55 °C/0.1 mbar for 24 h in B-585 oven (Buchi Glass Drying Oven, Switzerland) filled with P_2O_5. Yield: 5.00 g (81.6%). M_n = 72000 g mol^{-1}, M_w/M_n = 1.50 (by GPC); T_g = -46 °C (by DSC); T_{onset} = 240°C (by TGA in air); σ_{DC} = 9.9 × 10^{-6} Sm cm^{-1} (30 °C); ^{1}H NMR (600.2 MHz, DMSO-d_6) δ(ppm): 9.07 (s, 1H), 7.74 (s, 1H), 7.68 (s, 1H), 4.16 (t, J = 7.2 Hz, 2H), 4.01 (br.s., 4H), 3.85 (s, 3H), 3.80 – 3.42 (m, 39H), 3.24 (s, 3H), 3.00 (s, 2H), 2.19 – 1.31 (m, 6H), 1.77 (p, J = 7.4 Hz, 2H), 1.27 (sext, *J* = 7.4 Hz, 2H), 1.25 – 0.45 (m, 6H), 0.90 (t, *J* = 7.4 Hz, 3H) (for the detailed assignment see **Figure S10**, the ratio between ILM and PEGM in PIL^{-} was determined by ^{1}H NMR as 1:1.2 by mol); ^{13}C NMR (150.9 MHz, DMSO-d_6) δ(ppm): 176.7, 136.5, 123.6, 122.2 120.1 (q, J_{CF} = 324.3 Hz), 71.3, 69.8, 69.56, 67.8, 63.8, 58.0, 51.1, 48.5, 35.7, 31.3, 23.01, 18.7, 16.6, 13.2 (**Figure S11**); ^{19}F NMR (564.7 MHz, DMSO-d_6) δ(ppm): -79.8 (s) (**Figure S12**); IR (ATR-mode): 3151 (w), 3111 (w), 2871 (s, ν_{CH}), 1727 (s, $\nu_{C=O}$), 1572 (w), 1454 (m, ν_{CH}), 1387 (w), 1351 (w), 1322 (s, ν_{asSO2}), 1298 (m), 1248 (m, $\nu_{as\text{-}C\text{-}O\text{-}C\text{-}}$), 1224 (m, ν_{CF}), 1177 (vs, ν_{sSO2}), 1120 (vs, $\nu_{\text{-}C\text{-}O\text{-}}$), 1054 (s, ν_{CF}), 949 (m), 852 (m), 621 (s) cm^{-1} (**Figure S13**).

Determination of the ratio between ILM and PEGM in PIL⁻.

The proportion of ILM (X_{ILM}) and PEGM (X_{PEGM}) units in PIL⁻ was determined by ^{1}H NMR in isolated copolymer sample using the integrals of the signals at 3.42 ppm (-O-C<u>H</u>$_3$ from PEGM, see 7 on **Figure S10**) and at 3.00 ppm (-C<u>H</u>$_2$-SO_2-N-SO_2CF_3 from ILM, see 6 on **Figure S10**) and the following equations:

$$X_{\mathrm{ILM}} = \frac{(6)}{n(\mathrm{H})} \quad \text{(eq S8)}$$

$$X_{\mathrm{PEGM}} = \frac{(7)}{n(\mathrm{H})} \quad \text{(eq S9)}$$

$$[\mathrm{PEGM}]:[\mathrm{ILM}] = X_{\mathrm{PEGM}}/X_{\mathrm{ILM}} \quad \text{(eq S10)}$$

, where n(H) is the number of hydrogens in the group.

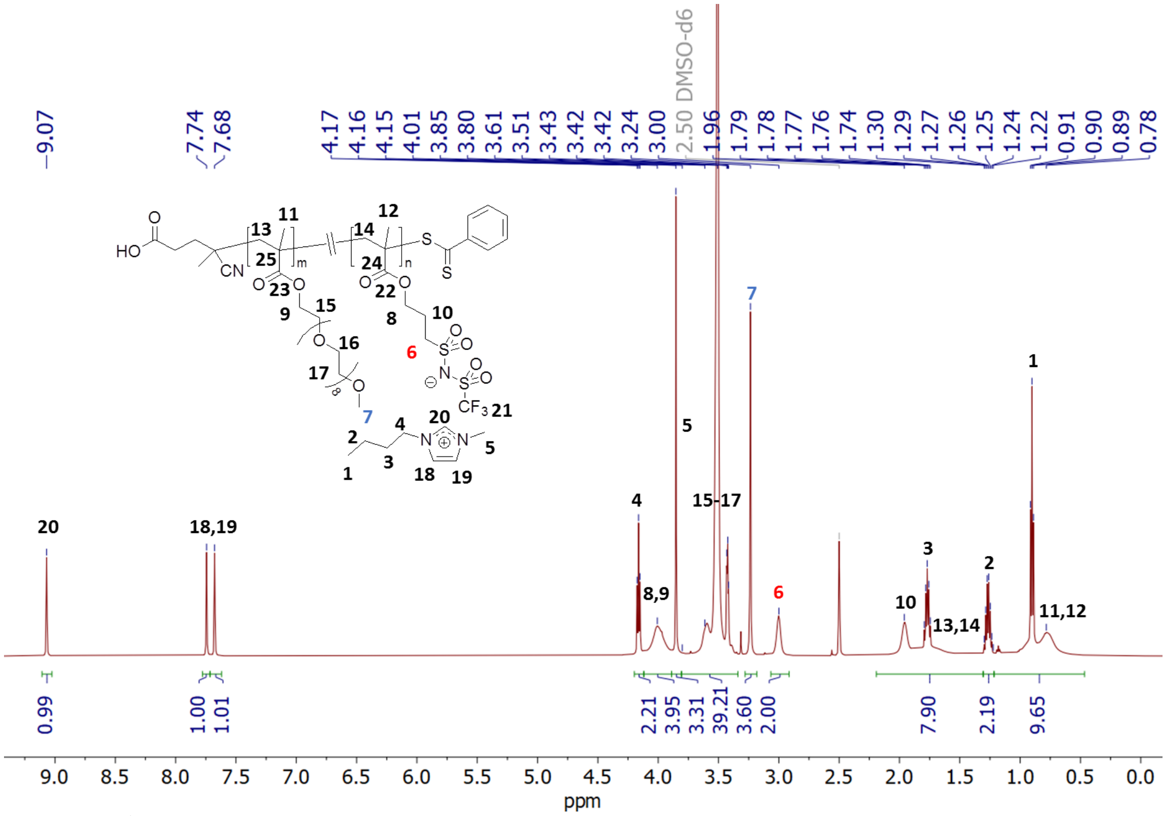

Figure S10. ^{1}H NMR of polymethacrylate/imidazolium/TFSI-based anionic PIL⁻ (DMSO-d_6, 25 °C).

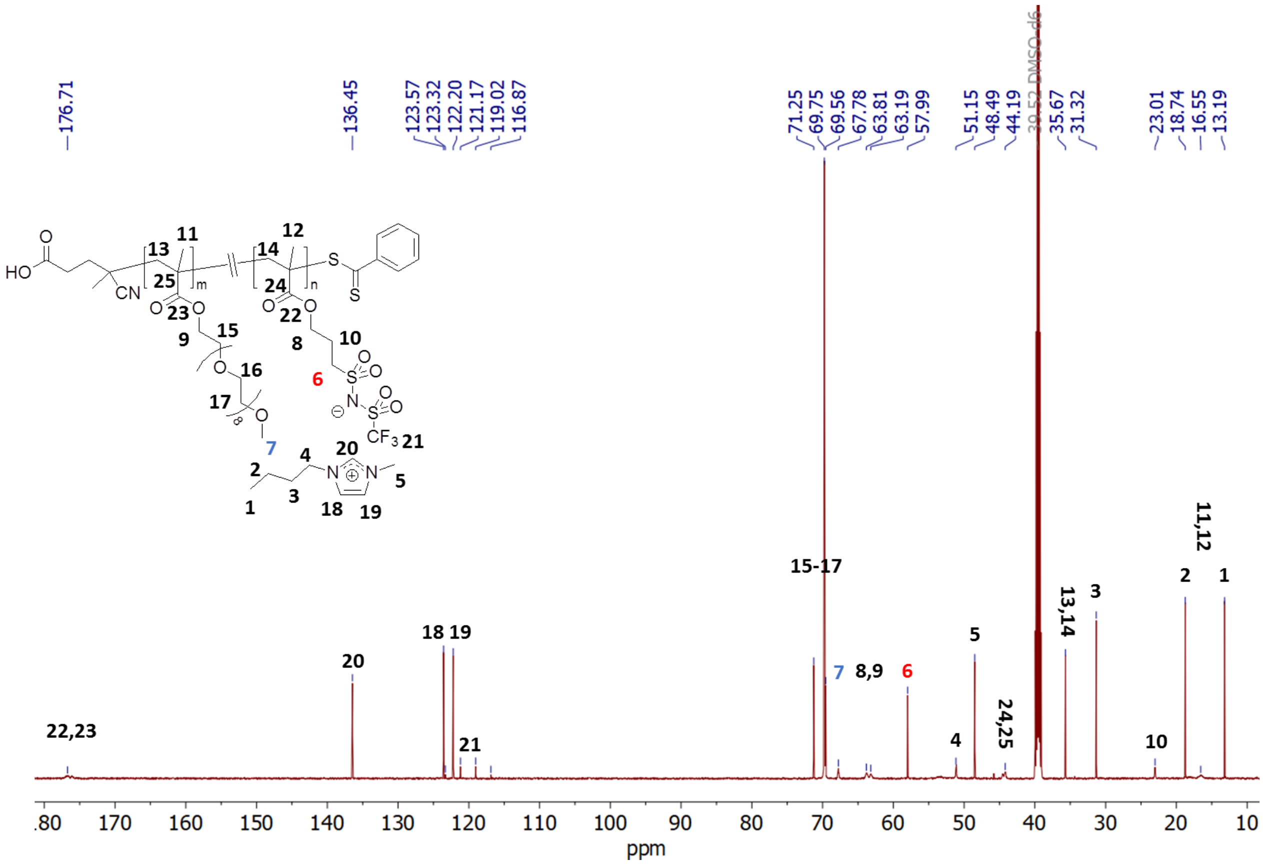

Figure S11. ^{13}C NMR of polymethacrylate/imidazolium/TFSI-based anionic PIL^{-} (DMSO-d_6, 25 °C).

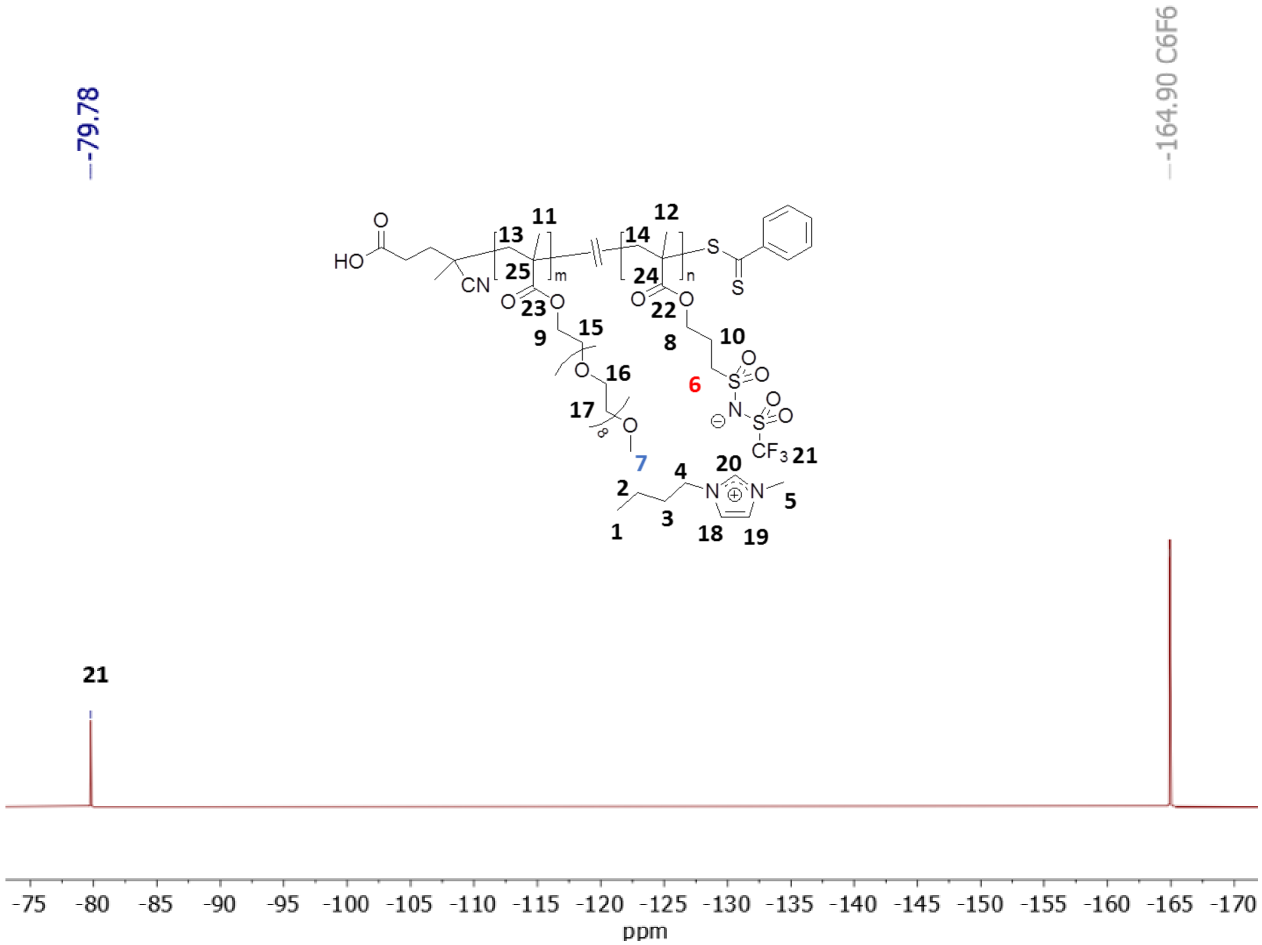

Figure S12. ^{19}F NMR of polymethacrylate/imidazolium/TFSI-based anionic PIL^{-} (DMSO-d_6, 25 °C).

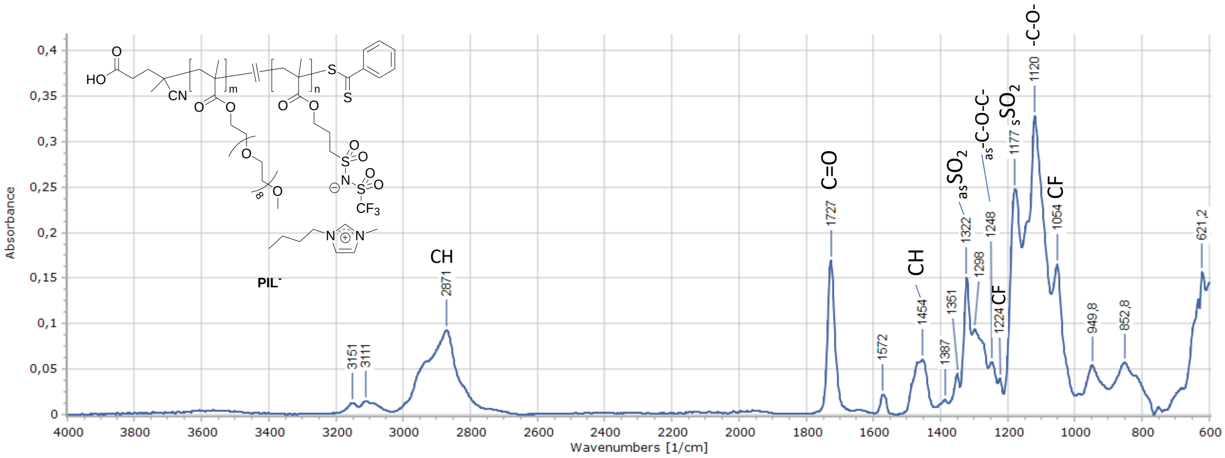

Figure S13. FTIR spectrum of polymethacrylate/imidazolium/TFSI-based anionic PIL^{-}.

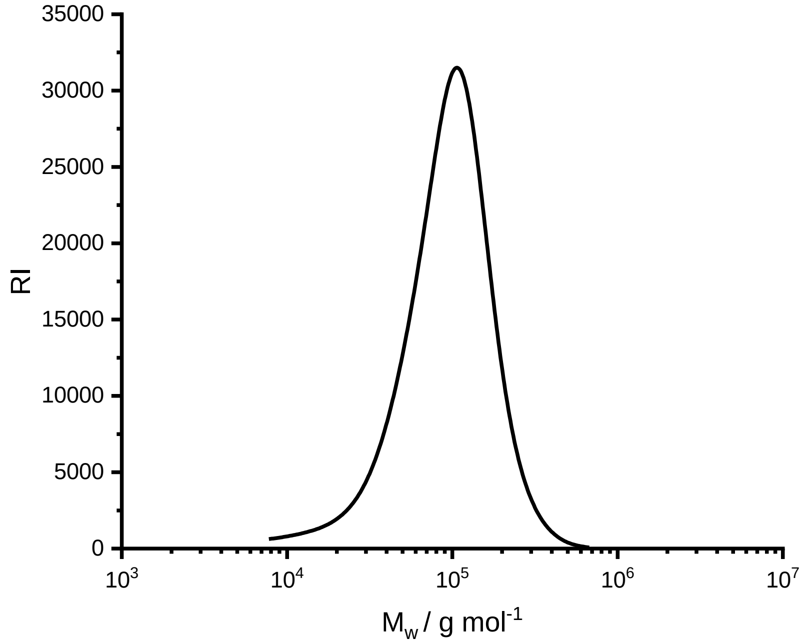

Figure S14. SEC traces (50 °C, 0.1 M LiTFSI in DMF, calibration with PMMA standards) of polymethacrylate/imidazolium/TFSI-based anionic PIL$^-$.

IV. Preparation of dynamic ion gels (DIGs)

General procedure for the preparation of DIGs. Complex coacervates were prepared in stoichiometric conditions regarding ion pairs from cationic PIL$^+$ (33 wt%) and anionic PIL$^-$ (67 wt%). A solution of PIL$^+$ (125 mg, 0.241 mmol of ion pairs) in 1.0 mL of acetone was added drop-wise into a solution of PIL$^-$ (260 mg, 0.241 mmol of ion pairs) in 1.0 mL of acetone under constant stirring. The formation of a turbid suspension was observed immediately. The resulting suspension, having a total polymer concentration of ~ 20 wt%, was vortex mixed for 1 min to improve mixing quality. Acetone was evaporated under reduced pressure and the resulting DIG was annealed for 24 h at 70 °C/1 mm Hg.

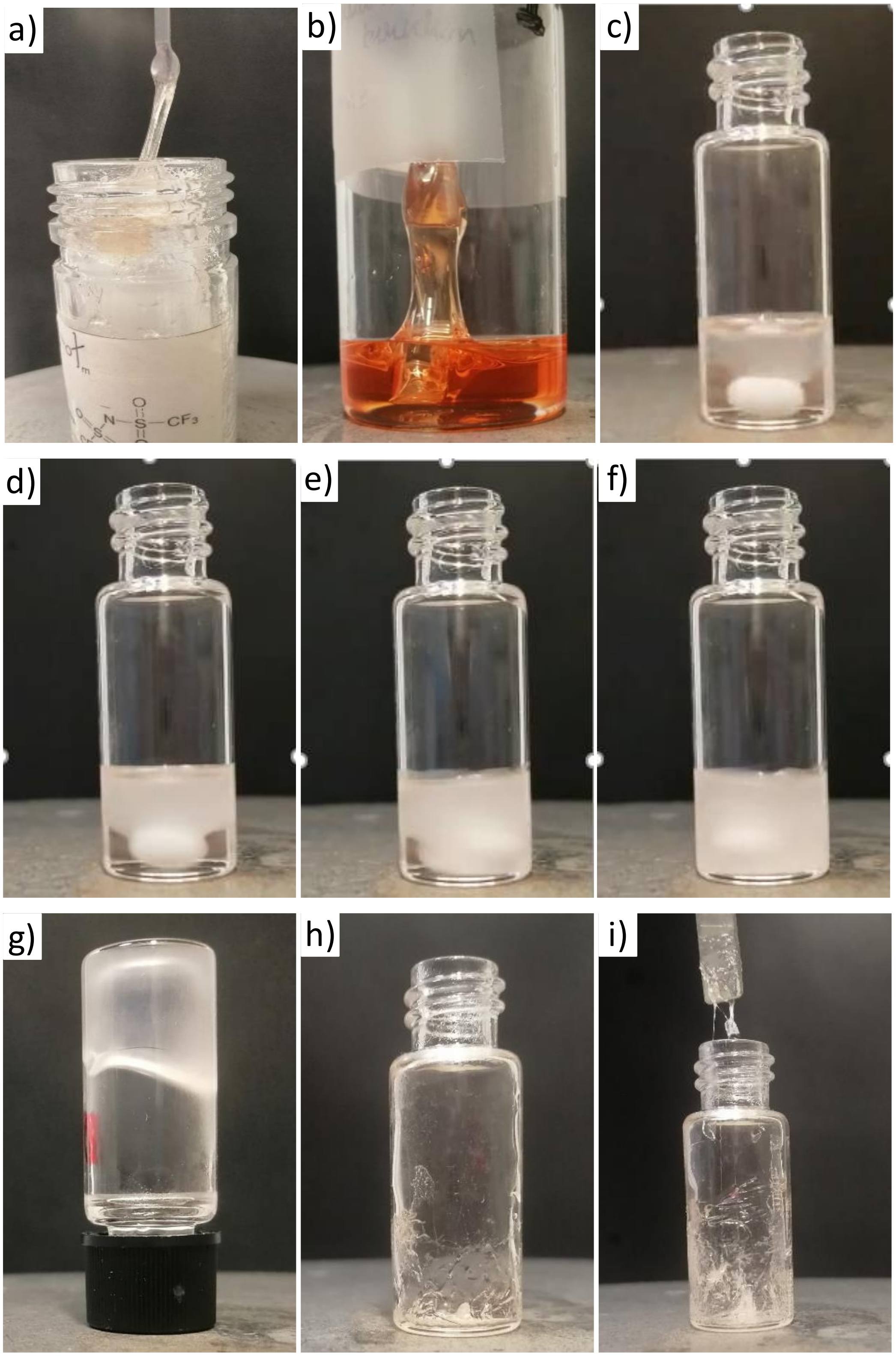

Figure S15. Optical images of PIL^+ (a, elastic solid), PIL^- (b, viscous liquid), solid like coacervate (c to g) and the resulting DIG (h and i**,** elastic solid**)**.

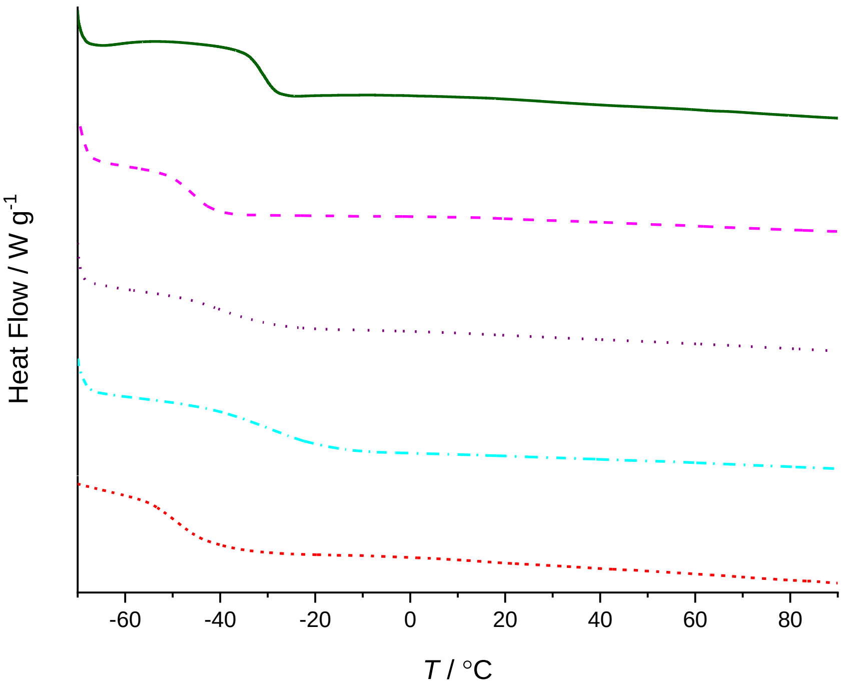

Figure S16. DSC curves (exo/up) of PIL^+ (green solid line), PIL^- (pink dashed line), DIG (purple dotted line), DIG_{ext} (blue dashed-dotted line) and DIG_{IL} (red short dashed line).

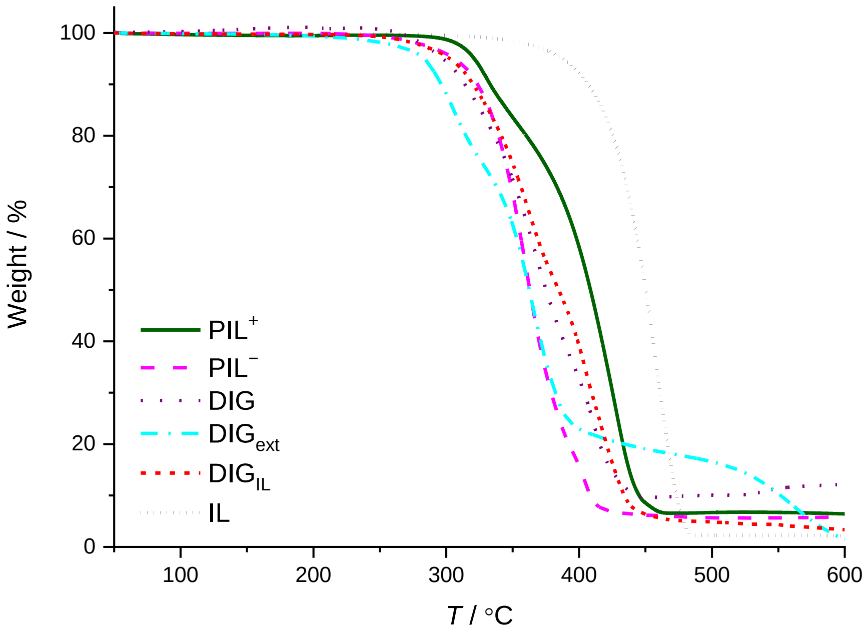

Figure S17. TGA curves of PIL^+ (green solid line), PIL^- (pink dashed line), DIG (purple dotted line), DIG_{ext} (blue dashed-dotted line), DIG_{IL} (red short dashed line) and IL (black short dotted line).

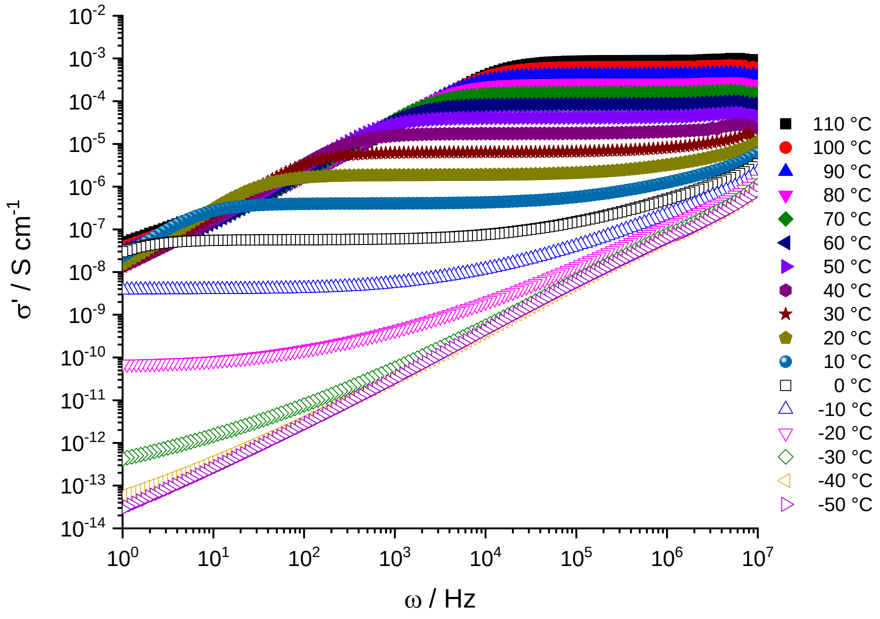

Figure S18. Frequency dependence of ionic conductivity σ' measured by BDS from 110 to –50 °C for PIL^{+}.

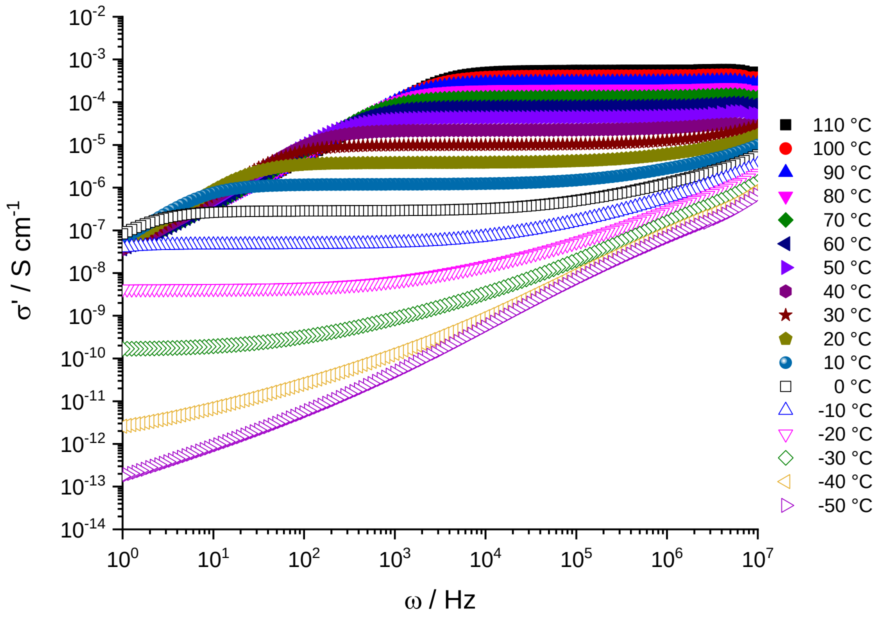

Figure S19. Frequency dependence of ionic conductivity σ' measured by BDS from 110 to –50 °C for PIL^{-}.

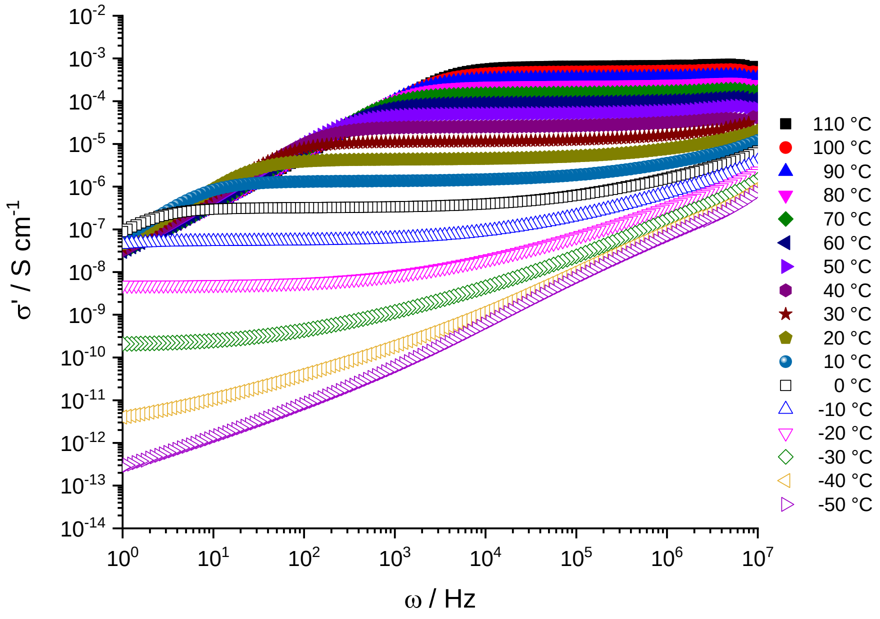

Figure S20. Frequency dependence of ionic conductivity σ’ measured by BDS 110 to –50 °C for DIG.

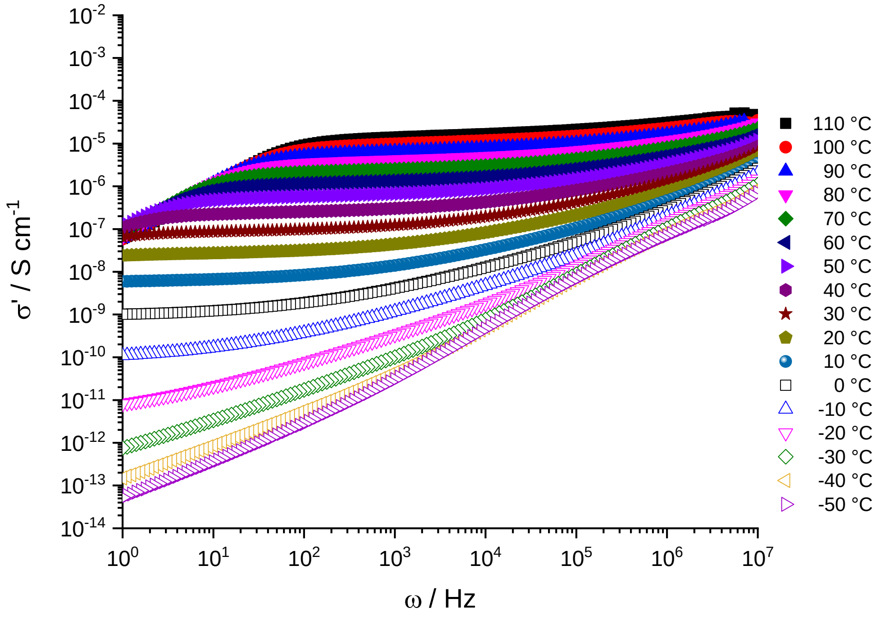

Figure S21. Frequency dependence of ionic conductivity σ’ measured by BDS from 110 to –50 °C for DIG_{ext}.

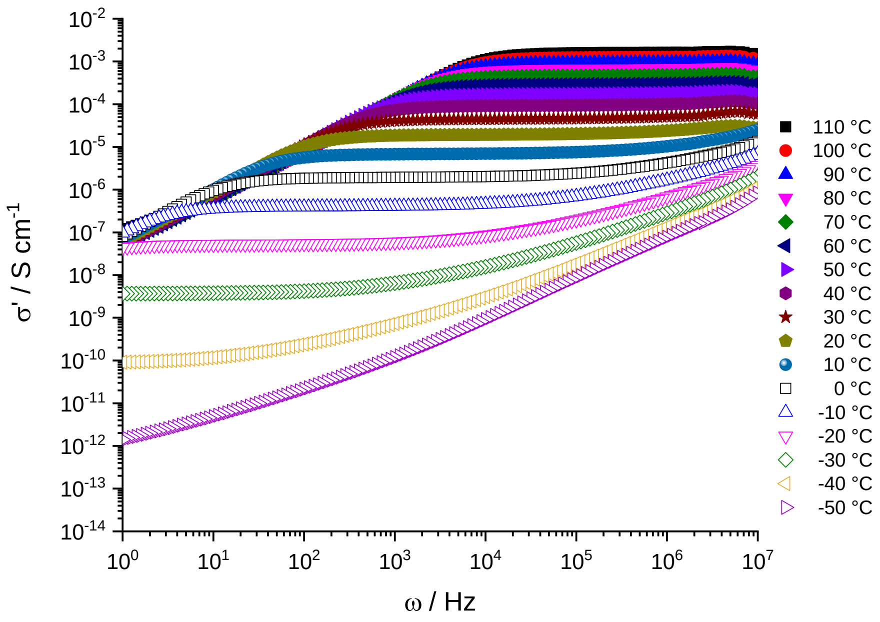

Figure S22. Frequency dependence of ionic conductivity σ' measured by BDS from 110 to –50 °C for DIG_{IL}.

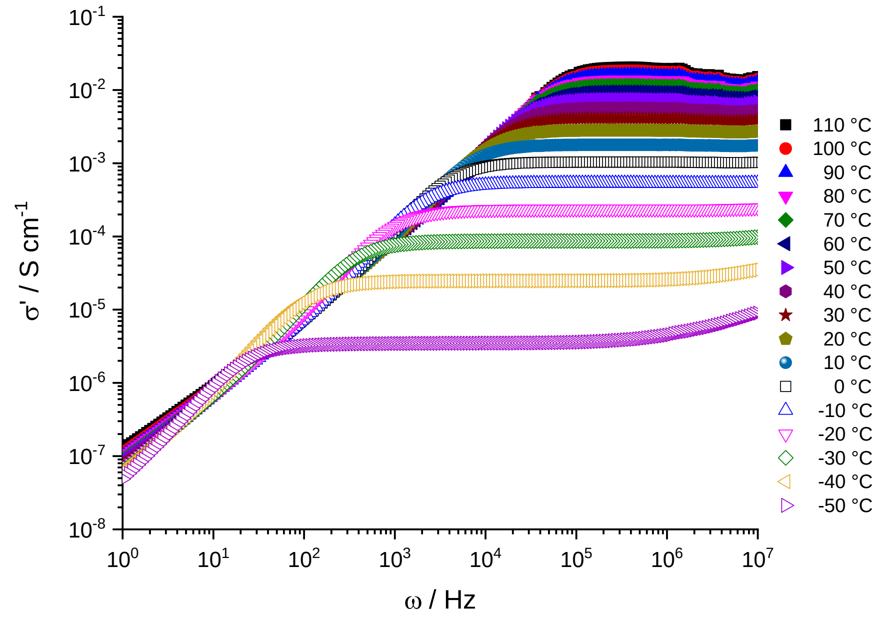

Figure S23. Frequency dependence of ionic conductivity σ' measured by BDS from 110 to –50 °C for IL.

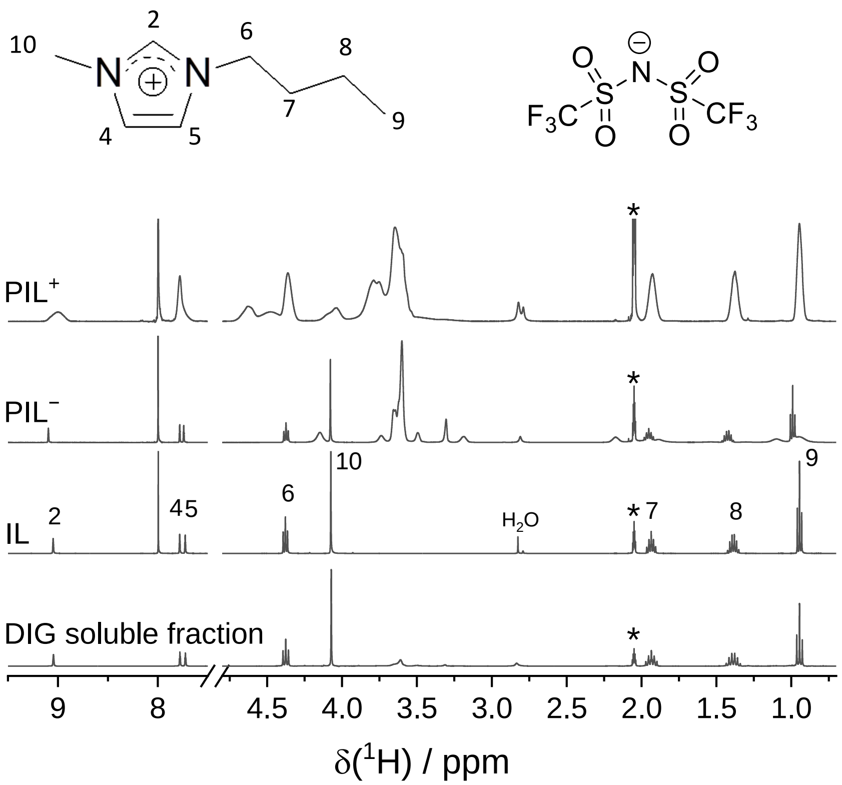

Figure S24. ^{1}H NMR (acetone-*d*$_6$, 25 °C) of PIL$^+$, PIL$^-$, [C_4C_1Im][TFSI] IL and the extracted material from solvent extraction experiment. * Stands for the ^{1}H signal of acetone (δ = 2.05 ppm).

V. Determination of the amount of released ionic liquid

Preparation of DIG$_{ext}$ by solvent extraction of DIG and quantification of released IL by gravimetry. A solution of PIL$^+$ (125 mg, 0.241 mmol of ion pairs) in 1.0 mL of acetone was added drop-wise into a solution of PIL$^-$ (260 mg, 0.241 mmol of ion pairs) in 1.0 mL of acetone under constant stirring to yield the solution with stoichiometrically charge-matched 1:1 ratio and having a total polymer concentration of ~ 20 wt%. The formation of the turbid suspension is observed immediately. The resulting suspension was vortex mixed for 1 min and further centrifuged (10000 rpm for 10 minutes at 15 °C) and the supernatant acetone solution was removed by decantation. 5 mL of fresh acetone were added to the residual coacervate, the suspension was vortex-mixed, centrifuged and the supernatant solution was again removed by decantation. This step was repeated

6 times. The extracted acetone layers were united and the solvent was evaporated under reduced pressure. A colorless liquid (94.9 mg) was obtained after drying for 24 h at 70 °C/1 mm Hg. This represents 24.6 wt% of the initial mass (i.e., PIL^+ and PIL^-) and generation of 93.9 mol% of free ion pairs according to the initial content of ion pairs in PIL^+ and PIL^-. ^{1}H NMR (500.2 MHz, acetone-d_6) δ(ppm): see **Figure S24**.

In-situ quantification of released IL during DIG formation by ^{19}F NMR spectroscopy.

Quantification of released IL was performed by quantitative ^{19}F NMR spectroscopy using a solution of 1,4-bis(trifluoromethyl)benzene (TFM2B, internal calibrant) and trichlorofluoromethane ($CFCl_3$, internal standard, δ = 0.00 ppm) in acetone-d_6. To minimize weighing uncertainties, a stock solution (denoted as solution A) of TFM2B (39.2 mg, 0.183 mmol) and $CFCl_3$ (34.3 mg, 0.25 mmol) was prepared in acetone-d_6 (4.9 mL). Then, a solution of PIL^+ (7.8 mg, 0.015 mmol of ion pairs) in 400 µL of solution A was added to a solution of PIL^- (16.1 mg, 0.015 mmol of ion pairs) in 300 µL of solution A inside an NMR tube. A whitening of the solution was readily observed and after vigorous stirring a white precipitate falling down to the bottom of the tube was formed (**Figure 3**). After taking a quantitative ^{19}F NMR spectra (**Figure S25**), the integrals of the internal calibrant (TFM2B) and the released IL were compared to the calculated mass of released IL (m_{IL}) during DIG formation using **equation S11**:

$$m_{\mathrm{IL}} = \frac{I_{\mathrm{IL}} \times C_{\mathrm{TFM2B}}}{I_{\mathrm{TFM2B}}} \times M_{\mathrm{IL}} \times V \qquad \text{(eq S11)}$$

with I_{IL} the integral of the signal of IL at δ(^{19}F) = -78.828 ppm, I_{TFM2B} the integral of the signal of TFM2B at δ(^{19}F) = -62.679 ppm, C_{TFM2B} the concentration of TFM2B in solution A (C_{TFM2B} = 37.35 mM), M_{IL} the molar mass of IL and V the volume of the solution of the PIL^+ and PIL^- mixture. In the experiment described herein m_{IL} = 5.7 mg. This corresponds to 23.8 wt% of the initial mass

of PIL$^+$ and PIL$^−$, and it accounts for the generation of 90.8 mol% of free ion pairs according to the initial content of ion pairs in PIL$^+$ and PIL$^−$.

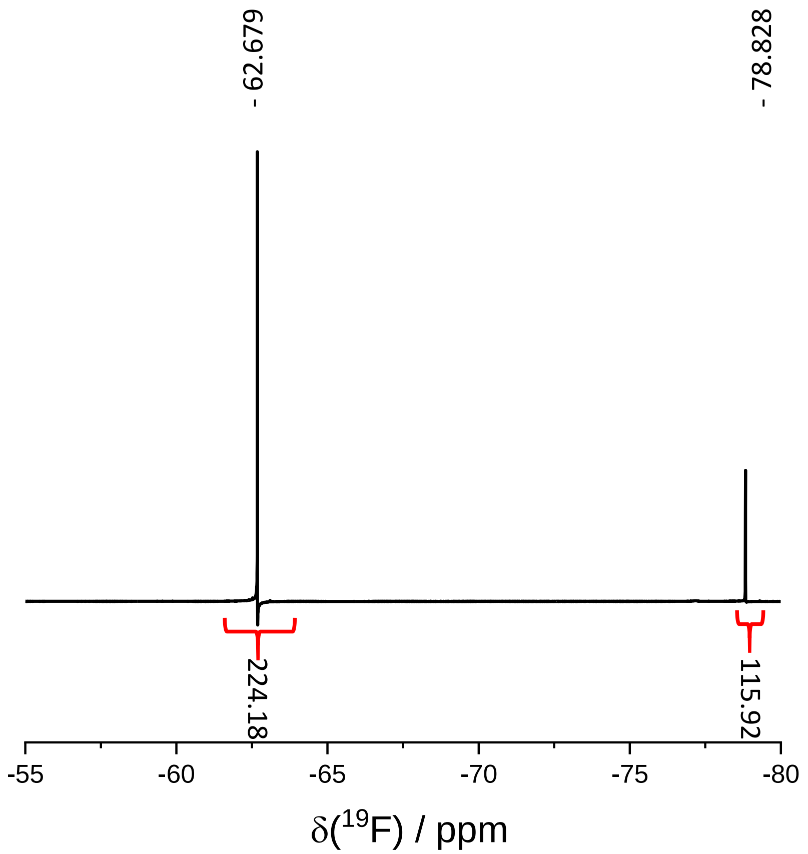

Figure S25. ^{19}F NMR of DIG prepared in acetone-d_6 inside the NMR tube containing fluorinated internal references 1,4-bis(trifluoromethyl)benzene and $CFCl_3$.

Preparation of IL-doped DIG$_{IL}$. Aiming at an IL-doped DIG containing 40 wt% of IL and considering the quantification of IL released after DIG formation by ^{19}F NMR (i.e., 23.8 wt%), we have prepared DIG$_{IL}$ as follows. A solution of PIL$^+$ (57 mg, 0.11 mmol of ion pairs) in acetone (1.0 mL) was added drop-wise to a stirred solution of IL (47 mg, 0.11 mmol) and anionic PIL$^−$ (118 mg, 0.11 mmol of ion pairs) in acetone (1.0 mL). The formation of a turbid suspension was observed immediately and the complex coacervate suspension was additionally vortexed for 30 – 45 s to ensure the completeness of ionic exchanges between oppositely charged PILs. Acetone was

removed by rotary evaporation and the obtained sample was annealed under vacuum for 24 h at 70 °C/1 mm Hg. In order to quantify the total content of released IL during the preparation of IL-doped DIG_{IL}, we have performed a quantitative ^{19}F NMR spectroscopy using a solution A of TFM2B and $CFCl_3$, in acetone-d_6, as explained above. Briefly, a solution of PIL^+ (3.0 mg, 0.0059 mmol of ion pairs) in 300 µL of solution A was added to a solution of solution of IL (2.5 mg, 0.0060 mmol) and $PIL^–$ (6.3 mg, 0.0059 mmol of ion pairs) in 300 µL of solution A inside an NMR tube. After taking a quantitative ^{19}F NMR spectra, the integrals of the internal calibrant (TFM2B) and the total content of released IL were compared to calculate the mass of released IL (m_{IL}) during DIG_{IL} formation. Experimentally, we have obtained m_{IL} = 3.05 mg. which corresponds to ~33 wt% of the initial mass of PIL^+ and $PIL^–$.

VI. Tables

Table S1. Parameters obtained by WLF fits of the rheology data.

sample	*$T_0 = T_g + 40$ K*		
	C_1 / ℃	*C_2 / ℃*	*T_0 / ℃*
PIL^+	4.3	42.9	10
$PIL^–$	7.5	81.3	-6
DIG	10.3	173.1	1
DIG_{ext}	8.3	109.1	10
DIG_{IL}	9.1	165.3	-9

Table S2. Parameters obtained by VFT fits of the BDS data.

sample	σ_∞ / S cm^{-1}	*B* / *K*	T_{VFT} / *K*	$T_g - T_{VFT}$ / *K*	σ_{DC} at 30 °C / S cm^{-1}
PIL^+	0.31	1040	206	37	6.4×10^{-6}
$PIL^–$	0.18	1108	190	37	9.9×10^{-6}
DIG	0.26	1154	188	46	1.2×10^{-5}
DIG_{ext}	0.02	1388	191	52	9.0×10^{-8}
DIG_{IL}	0.38	1066	185	39	4.5×10^{-5}

Table S3. Overview of the most highly conductive PILs and samples from this work reported to date (updated on February, 2024).

N°	Chemical structure	Ionic conductivity		M_n[c] kDa	Physical state of PIL (mechanical properties)	Ref.
		σ_{DC}[a] S cm^{-1}	T[b] °C			
1	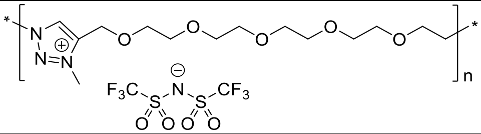	1.2×10^{-4}	30	8.2[d]	amber-colored low MM viscous material (n.d.)[f]	5
2	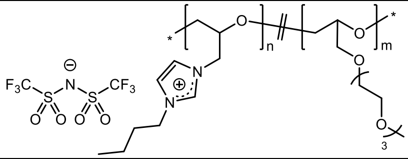	1.2×10^{-4}	25	5.4	cold flowing viscous liquid (n.d.)	6
3	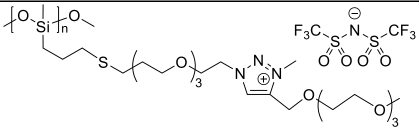	6.7×10^{-5}	30	30.2	yellow viscous liquid (n.d.)	7
4	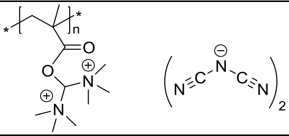	5.5×10^{-5}	25	1830[d]	colorless coatings on metals, brittle and opaque self-standing films (n.d.)	8
5	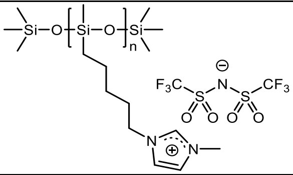	5.0×10^{-5}	25	2.4[d]	Brown oil (dynamic shear loss modulus G″ (rheology, -30oC): 130 MPa)	9
6	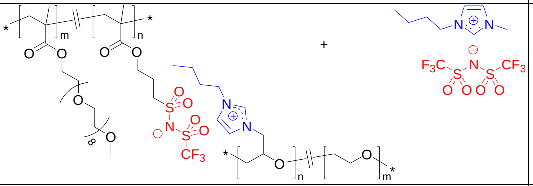	4.5×10^{-5}	30	-	**This work, DIG$_{IL}$ (with a total IL content of 33 wt%)**	**This work**
7	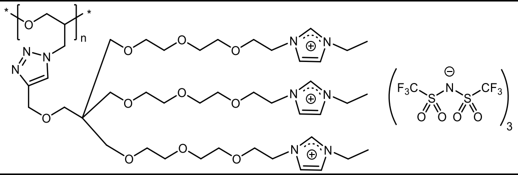	3.6×10^{-5}	25	1900[d]	cold flowing viscous mass (n.d.)	10
8	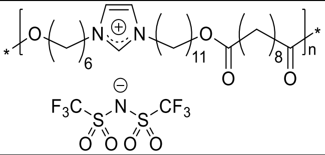	3.2×10^{-5}	25	26.0	clear viscous oil (n.d.)	11
9	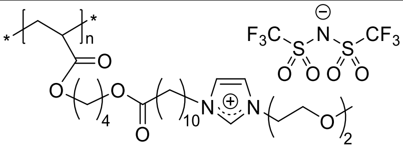	2.8×10^{-5}	25	6.7	(n.d.)	12

10	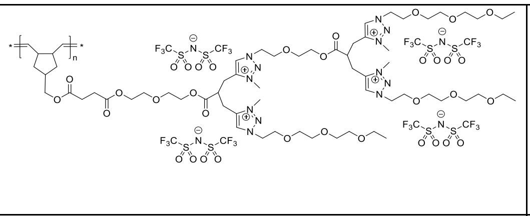	2.4×10^{-5}	30	23.0	from purple sticky soft solid to cross-linked soft self-standing films (maximum stress and the elongation at break (tensile testing, 25ºC): 0.17 MPa and 170%)	13
11	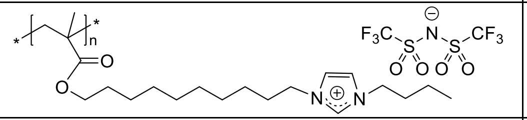	2.5×10^{-5}	30	26.8	yellow viscous oil (n.d.)	14
12	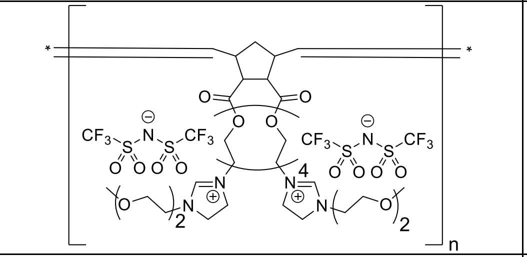	2.3×10^{-5}	25	45.0	viscous liquid (n.d.)	15
13	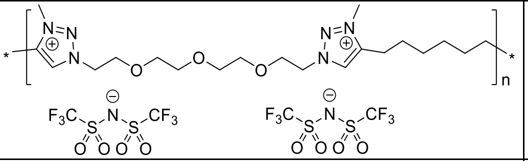	1.8×10^{-5}	25	71.7	brown gummy sticky solid (n.d.)	16
14	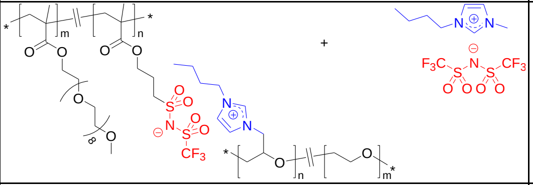	1.2×10^{-5}	30	-	**This work, DIG**	**This work**
15	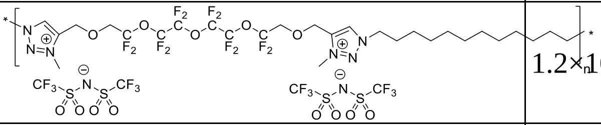	1.2×10^{-5}	30	-	brown viscous oil (n.d.)	17
16	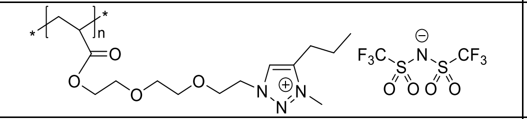	1.1×10^{-5}	30	113.6	orange viscous material (n.d.)	18
17	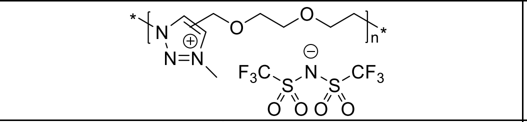	1.0×10^{-5}	25	44.0	dark brown viscous gummy solid (n.d.)	19
18	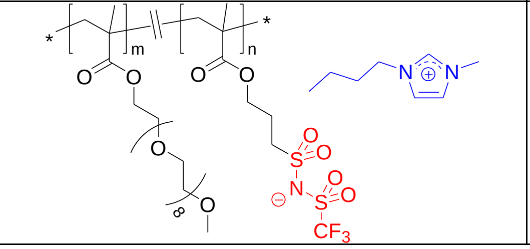	9.9×10^{-6}	30	72.4	**This work, PIL^{-}**	**This work**

19	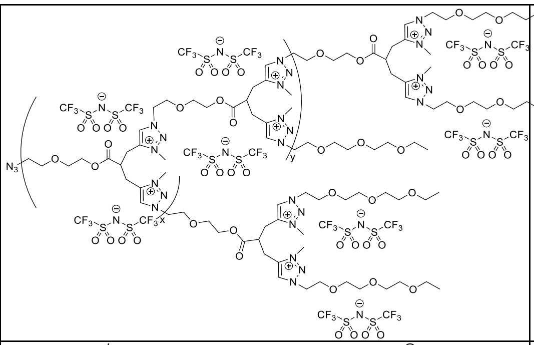	7.7×10^{-6}	30	252[d]	yellow viscous oil (n.d.)	**20**
20	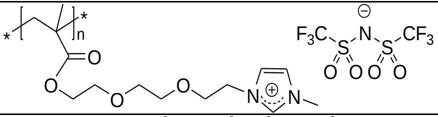	6.9×10^{-6}	25	194	transparent cold flowing viscous mass (n.d.)	21
21	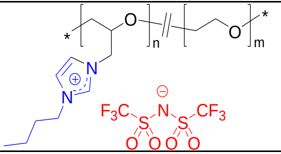	6.4×10^{-6}	30	550	**This work, PIL^+**	**This work**
22	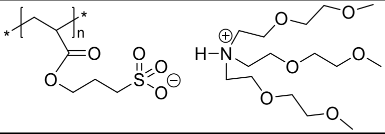	6.3×10^{-6}	27	99.5	(n.d.)	22
23	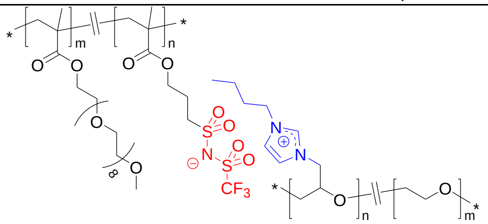	9.0×10^{-8}	30	-	**This work, DIG_{ext}**	**This work**

[a]DC conductivity under anhydrous conditions at 25 or 30°C; [b]Temperature at which σ_{DC} was measured; [c]Number-average molar mass; [d]Number-average molar mass measured for neutral precursor; [e]Weight-average molar mass. [f]N.d.=not determined.

VII. References

1. Porcarelli, L.; Shaplov, A. S.; Salsamendi, M.; Nair, J. R.; Vygodskii, Y. S.; Mecerreyes, D.; Gerbaldi, C., Single-ion block copoly (ionic liquid) s as electrolytes for all-solid state lithium batteries. *ACS Appl. Mater. Interfaces* **2016,** *8* (16), 10350-10359, DOI: 10.1021/acsami.6b01973.
2. Shaplov, A.; Vlasov, P.; Armand, M.; Lozinskaya, E.; Ponkratov, D.; Malyshkina, I.; Vidal, F.; Okatova, O.; Pavlov, G.; Wandrey, C., Design and synthesis of new anionic "polymeric ionic liquids" with high charge delocalization. *Polym. Chem.* **2011,** *2* (11), 2609-2618, DOI: 10.1039/c1py00282a.
3. Menges, F. Spectragryph-Optical Spectroscopy Software, 1.2.15.2020. Available Online: Http://Www.Effemm2.de/Spectragryph (Accessed on 12 May 2021).
4. Williams, M. L.; Landel, R. F.; Ferry, J. D., The temperature dependence of relaxation mechanisms in amorphous polymers and other glass-forming liquids. *J. Am. Chem. Soc.* **1955,** *77* (14), 3701-3707, DOI: 10.1021/JA01619A008.
5. Puguan, J. M. C.; Boton, L. B.; Kim, H., Triazole-based ionene exhibiting tunable structure and ionic conductivity obtained via cycloaddition reaction: A new polyelectrolyte for electrochromic devices. *Sol. Energy Mater. Sol. Cells* **2018,** *188*, 210-218, DOI: 10.1016/j.solmat.2018.09.009.
6. Hu, H.; Yuan, W.; Jia, Z.; Baker, G. L., Ionic liquid-based random copolymers: a new type of polymer electrolyte with low glass transition temperature. *RSC Adv.* **2015,** *5* (5), 3135-3140, DOI: 10.1039/C4RA13432J.
7. Jourdain, A.; Serghei, A.; Drockenmuller, E., Enhanced ionic conductivity of a 1, 2, 3-triazolium-based poly (siloxane ionic liquid) homopolymer. *ACS Macro Lett.* **2016,** *5* (11), 1283-1286, DOI: 10.1021/acsmacrolett.6b00761.
8. Shaplov, A. S.; Lozinskaya, E. I.; Losada, R.; Wandrey, C.; Zdvizhkov, A. T.; Korlyukov, A. A.; Lyssenko, K. A.; Malyshkina, I. A.; Vygodskii, Y. S., Polymerization of the new double‐charged monomer bis‐1, 3 (N, N, N‐trimethylammonium dicyanamide)‐2‐propylmethacrylate and ionic conductivity of the novel polyelectrolytes. *Polym. Adv. Technol.* **2011,** *22* (4), 448-457, DOI: 10.1002/pat.1569.
9. Wojnarowska, Z.; Feng, H.; Fu, Y.; Cheng, S.; Carroll, B.; Kumar, R.; Novikov, V. N.; Kisliuk, A. M.; Saito, T.; Kang, N.-G., Effect of chain rigidity on the decoupling of ion motion from segmental relaxation in polymerized ionic liquids: Ambient and elevated pressure studies. *Macromolecules* **2017,** *50* (17), 6710-6721, DOI: 10.1021/acs.macromol.7b01217.
10. Ikeda, T., Poly (ionic liquid) s with branched side chains: polymer design for breaking the conventional record of ionic conductivity. *Polym. Chem.* **2021,** *12* (5), 711-718, DOI: 10.1039/D0PY01333A.
11. Lee, M.; Choi, U. H.; Salas‐de la Cruz, D.; Mittal, A.; Winey, K. I.; Colby, R. H.; Gibson, H. W., Imidazolium polyesters: structure–property relationships in thermal behavior, ionic conductivity, and morphology. *Adv. Funct. Mater.* **2011,** *21* (4), 708-717, DOI: 10.1002/adfm.201001878.
12. Lee, M.; Choi, U. H.; Colby, R. H.; Gibson, H. W., Ion conduction in imidazolium acrylate ionic liquids and their polymers. *Chem. Mater.* **2010,** *22* (21), 5814-5822, DOI: 10.1021/cm101407d.
13. Li, H.; Zhang, H.; Liao, X.; Sun, R.; Xie, M., Incorporating trifunctional 1, 6-heptadiyne moiety into polyacetylene ionomer for improving its physical and conductive properties. *Polym. Chem.* **2020,** *11* (19), 3322-3331, DOI: 10.1039/D0PY00109K.

14. Chen, M.; Dugger, J. W.; Li, X.; Wang, Y.; Kumar, R.; Meek, K. M.; Uhrig, D. W.; Browning, J. F.; Madsen, L. A.; Long, T. E., Polymerized ionic liquids: Effects of counter ‐ anions on ion conduction and polymerization kinetics. *J. Polym. Sci. Part A: Polym. Chem.* **2018,** *56* (13), 1346-1357, DOI: 10.1002/pola.29015.
15. Price, T. L., Jr.; Choi, U. H.; Schoonover, D. V.; Wang, D.; Heflin, J. R.; Xie, R.; Colby, R. H.; Gibson, H. W., Studies of Ion Conductance in Polymers Derived from Norbornene Imidazolium Salts Containing Ethyleneoxy Moieties. *Macromolecules* **2019,** *52* (4), 1389-1399, DOI: 10.1021/acs.macromol.8b02303.
16. Cotessat, M.; Flachard, D.; Nosov, D.; Lozinskaya, E. I.; Ponkratov, D. O.; Schmidt, D. F.; Drockenmuller, E.; Shaplov, A. S., Effects of repeat unit charge density on the physical and electrochemical properties of novel heterocationic poly (ionic liquid) s. *New J. Chem.* **2021,** *45* (1), 53-65, DOI: 10.1039/D0NJ04143B.
17. Anaya, O.; Kallel Elloumi, A.; Thankappan, H.; Abdelhedi Miladi, I.; Serghei, A.; Ben Romdhane, H.; Drockenmuller, E., Synthesis and Structure/Properties Correlations of Fluorinated Poly (1, 2, 3-triazolium) s. *Chem. Afr* **2020,** *3*, 759-768, DOI: 10.1007/s42250-020-00164-1.
18. Sood, R.; Zhang, B.; Serghei, A.; Bernard, J.; Drockenmuller, E., Triethylene glycol-based poly (1, 2, 3-triazolium acrylate) s with enhanced ionic conductivity. *Polym. Chem.* **2015,** *6* (18), 3521-3528, DOI: 10.1039/C5PY00273G.
19. Colliat-Dangus, G.; Obadia, M. M.; Vygodskii, Y. S.; Serghei, A.; Shaplov, A. S.; Drockenmuller, E., Unconventional poly (ionic liquid) s combining motionless main chain 1, 2, 3-triazolium cations and high ionic conductivity. *Polym. Chem.* **2015,** *6* (23), 4299-4308, DOI: 10.1039/C5PY00526D.
20. Wu, J.; Chen, J.; Wang, J.; Liao, X.; Xie, M.; Sun, R., Synthesis and conductivity of hyperbranched poly (triazolium) s with various end-capping groups. *Polym. Chem.* **2016,** *7* (3), 633-642, DOI: 10.1039/C5PY01735A.
21. Shaplov, A. S.; Ponkratov, D. O.; Aubert, P.-H.; Lozinskaya, E. I.; Plesse, C.; Maziz, A.; Vlasov, P. S.; Vidal, F.; Vygodskii, Y. S., Truly solid state electrochromic devices constructed from polymeric ionic liquids as solid electrolytes and electrodes formulated by vapor phase polymerization of 3, 4-ethylenedioxythiophene. *Polymer* **2014,** *55* (16), 3385-3396, DOI: j.polymer.2014.04.013.
22. Frenzel, F.; Guterman, R.; Anton, A. M.; Yuan, J.; Kremer, F., Molecular dynamics and charge transport in highly conductive polymeric ionic liquids. *Macromolecules* **2017,** *50* (10), 4022-4029, DOI: 10.1021/acs.macromol.7b00554.
